# Prediction of Overall Survival in Patients With Oesophageal Cancer Using AI‐Based 3D CT Body Composition Analysis

**DOI:** 10.1002/jcsm.70363

**Published:** 2026-08-11

**Authors:** Christian Römer, Jens Hölzen, Andreas Pascher, Walter Heindel, Marc‐David Künnemann, René Hosch, Katarzyna Borys, Gesa Helen Pöhler

**Affiliations:** ^1^ Clinic for Radiology University Hospital Münster and University of Münster Münster Germany; ^2^ Department of General, Visceral and Transplant Surgery University Hospital Münster and University of Münster Münster Germany; ^3^ Institute of Diagnostic and Interventional Radiology and Neuroradiology University Hospital Essen Essen Germany; ^4^ Institute for Artificial Intelligence in Medicine (IKIM), University Hospital Essen Essen Germany

**Keywords:** artificial intelligence, body composition, computed tomography, myosteatosis, oesophageal cancer, sarcopenia

## Abstract

**Background:**

Oesophageal cancer remains the sixth most lethal malignancy, with 5‐year survival rates around 22%. CT‐derived 2D body composition analysis has emerged as a promising prognostic tool, but conventional, often manually created single‐slice L3 measurements are unsuited for future clinical implementation. We evaluated fully automated AI‐derived volumetric body composition indices as prognostic parameters using conventional L3 and BMI measurements as reference.

**Methods:**

This retrospective cohort study included patients with histologically confirmed oesophageal cancer treated between 2011 and 2024. Automated deep learning segmentation using the nnU‐Net‐based body and organ analysis pipeline quantified abdominal tissue volumes from staging CTs. Three normalized indices were calculated: sarcopenia index (SI, muscle/bone volume ratio), myosteatotic fat index (MFI, intramuscular/total adipose tissue volume ratio) and abdominal fat index (AFI, visceral/subcutaneous adipose tissue volume ratio). Cox proportional hazards models assessed prognostic value after sequential adjustment for age, sex, metastatic status, ECOG performance status, BMI and L3‐derived indices. Kaplan–Meier analysis evaluated survival differences stratified by sex‐specific median values.

**Results:**

The cohort comprised 563 patients (19.7% female), median age 65.4 years (IQR: 58.8–71.3); 10.1% (*n* = 57) had metastatic disease and 68.7% (*n* = 387) underwent surgery. Males had higher sarcopenia index (2.54 ± 0.41 vs. 2.24 ± 0.45, *p* < 0.001) and abdominal fat index (median 0.72 vs. 0.36, *p* < 0.001), whereas MFI showed no difference (*p* = 0.89). During median follow‐up of 22 months, 367 deaths (65.9%) occurred. Median overall survival was 28.6 months (95% CI: 23.7–33.3); 1‐year survival 70.7% (95% CI: 67.2%–74.8%) and 5‐year survival 32.8% (95% CI: 29.6%–38.3%), with marked differences by metastatic status (M0 vs. M1 1‐year survival: 73.4% vs. 46.4%). In the fully adjusted model incorporating all three volumetric indices alongside clinical covariates, BMI and L3‐derived parameters (*n* = 532), only sarcopenia index retained independent significance (HR = 0.56, 95% CI: 0.37–0.82, *p* = 0.003); all others showed *p* ≥ 0.26. Metastatic status (HR = 2.23, *p* < 0.001) and ECOG (HR = 1.29, *p* < 0.001) remained significant. All L3 indices lost significance alongside volumetric parameters (*p* > 0.14). Male patients with high sarcopenia index showed longer survival compared with patients with low sarcopenia index (33.3 vs. 21.8 months, *p* < 0.001); female patients showed larger descriptive contrasts (68.8 vs. 16.8 months, *p* < 0.001) that were formally confirmed by a significant sex‐SI Cox interaction (LRT *p* = 0.026).

**Conclusions:**

Automatic AI‐derived volumetric body composition parameters calculated from routine staging CTs predict overall survival in patients with oesophageal cancer and are associated with sex‐related differences of prognostic impact, supporting further evaluation toward future clinical use.

**Abbreviations**
AFI: abdominal fat index
AI: artificial intelligence
BCA: body composition analysis
BIA: bioelectrical impedance analysis
CT: computed tomography
DXA: dual‐energy X‐ray absorptiometry
ECOG: Eastern Cooperative Oncology Group (Performance Status)
IMATI: intermuscular adipose tissue index
MFI: myosteatotic fat index
OS: overall survival
SATI: subcutaneous adipose tissue index
SI: sarcopenia index
SMI: skeletal muscle index
VATI: visceral adipose tissue index

## 1 Introduction

Oesophageal cancer remains one of the most aggressive malignancies of the gastrointestinal tract, ranking as the seventh most common cancer worldwide and the sixth leading cause of cancer‐related mortality [1, 2]. Despite advances in multimodal treatment strategies combining neoadjuvant chemoradiotherapy, surgical resection and targeted therapies, the overall 5‐year survival rate remains poor: based on National Cancer Institute SEER data, the 5‐year relative survival rate for oesophageal cancer is 21.9% (2015–2021), ranging from 48.7% for localized disease to 5.4% for distant metastases [3]. This unfavourable prognosis underscores the critical need for improved risk stratification tools that can guide individualized treatment decisions and identify patients who may benefit from intensified therapeutic approaches [4].

Body composition analysis (BCA) has emerged as a promising avenue for prognostic assessment in oncology, with growing evidence linking imaging‐defined low muscle mass, myosteatosis and altered adipose tissue distribution to adverse outcomes across various malignancies [5, 6]. Consensus definitions of sarcopenia (EWGSOP2 [7], AWGS 2019 [8]) require a combination of low muscle strength, low appendicular muscle mass (assessed by dual‐energy X‐ray absorptiometry [DXA] or bioelectrical impedance analysis [BIA]) or reduced physical performance and are therefore not directly obtainable from staging CT. Collectively, traditional methods of body composition assessment, including BIA and DXA, require dedicated examinations and are not routinely performed in oncological staging workflows [5]. The BCA in oncology has nonetheless additively established CT‐derived measures of skeletal muscle and adipose tissue as imaging surrogates of muscle quantity and quality; these are biomarkers of body composition rather than diagnoses of guideline‐defined sarcopenia. CT imaging–defined low muscle mass has been extensively studied in oesophageal cancer patients and consistently associated with increased postoperative complications, reduced treatment tolerance and diminished survival [9, 10, 11], whereas the prognostic significance of myosteatosis, the pathological infiltration of skeletal muscle by adipose tissue, remains less well characterized [12, 13]. Myosteatosis has emerged as a novel biomarker in oncology, with meta‐analyses demonstrating that patients with myosteatosis have approximately 75% greater mortality risk compared with patients without this condition [14]. Similarly, the distribution of abdominal adipose tissue between visceral and subcutaneous compartments may reflect metabolic derangements and systemic inflammation that influence tumour biology and treatment response [15, 16]. CT imaging, universally employed for tumour staging, contains comprehensive information about skeletal muscle mass and adipose tissue compartments that can be extracted through systematic analysis [17, 18].

Recently, artificial intelligence (AI) and deep learning algorithms have revolutionized the quantification of body composition parameters from routine CT imaging [19, 20]. Automated segmentation tools now enable rapid, reproducible and observer‐independent extraction of tissue volumes, overcoming the limitations of manual or semi‐automated approaches that have historically hindered the clinical implementation of CT‐based BCA [21, 22]. The nnU‐Net framework has emerged as a particularly powerful self‐configuring method for deep learning‐based biomedical image segmentation, automatically adapting its preprocessing, network architecture, training and post‐processing for any new task without manual intervention [23]. This framework has been employed in combination with Total Segmentator [24] to develop comprehensive BCA tools such as the Body and Organ Analysis (BOA) pipeline, which combines tissue segmentation with organ segmentation to achieve a mean voxel body coverage of 93% ± 2% [25]. These AI‐driven pipelines facilitate the assessment of multiple tissue compartments, including visceral adipose tissue (VAT), subcutaneous adipose tissue (SAT), intramuscular adipose tissue (IMAT) and skeletal muscle, allowing for the calculation of composite indices that may capture distinct pathophysiological processes relevant to cancer prognosis [26, 27].

Künnemann et al. recently demonstrated in lung cancer patients that AI‐derived volumetric markers were associated with overall survival (OS), with the sarcopenia index (SI) outperforming conventional L3‐based skeletal muscle index measurements [28]. Similarly, Borys et al. validated volumetric body composition indices as independent prognostic factors for OS in melanoma patients, with findings replicated across internal and external validation cohorts [29]. However, BCA data in oesophageal cancer are rare.

Furthermore, the potential sex‐specific prognostic impact of these BCA parameters warrants systematic investigation, given the well‐documented sex differences in oesophageal cancer epidemiology and outcome [30, 31] alongside the marked sexual dimorphism in body composition. Evidence suggests that sex may be an independent prognostic factor in oesophageal carcinoma, with females generally demonstrating better survival outcomes, predominantly in squamous cell carcinoma [32, 33], though the underlying mechanisms remain incompletely understood.

The identification of reliable prognostic parameters is particularly relevant in the context of neoadjuvant therapy, where accurate risk stratification could guide treatment decisions and patient selection [34, 35]. Currently, clinical decision‐making regarding neoadjuvant treatment primarily relies on tumour stage and performance status, yet substantial heterogeneity in treatment response and survival outcomes persists among patients with similar clinicopathological characteristics [36].

The purpose of this study was to evaluate the prognostic impact of AI‐derived 3D BCA parameters in a large cohort of patients with oesophageal cancer undergoing routine staging CT imaging with conventional 2D L3 measurements as reference.

## 2 Methods

### 2.1 Study Design and Patient Selection

This retrospective cohort study analysed patients with oesophageal cancer treated at the University Hospital of Münster, Germany, between January 2011 and December 2024. Patients were eligible if they had histologically confirmed oesophageal cancer and a pretreatment staging CT meeting all of the following imaging criteria:

- complete abdominal coverage from the diaphragm to the pelvic floor,
- reconstructed slice thickness ≤ 5 mm and
- acquisition within ±60 days of histological diagnosis. CT acquisitions were agnostic to contrast agent and phase, and the field of view encompassed the abdominal cavity excluding the extremities.

Of 631 patients with oesophageal cancer diagnosed at our institution in the inclusion window, 68 patients were excluded for data inconsistency (*n* = 1), missing suitable CT imaging (*n* = 63) or missing follow‐up (*n* = 4), leaving 563 patients (452 male, 111 female) constituting the BCA cohort. Next, three analytic populations were distinguished:

- the BCA cohort (*n* = 563), used for descriptive statistics, sex comparisons and Kaplan–Meier survival analyses (Sections 3.2 and 3.4, 3.5);
- the L3 reference subset (*n* = 553, 98.2% of the BCA cohort), patients in whom the L3 vertebral level was captured on CT, used for the L3‐derived reference indices (Sections 2.3 and 3.3); and
- the complete case analytic cohort (*n* = 532), patients with complete L3 reference indices, BMI and ECOG performance status, used for the fully adjusted multivariable Cox models, the Δ*C* index analyses and the era sensitivity specifications (Sections 2.4 and 3.6).

**FIGURE 1:**
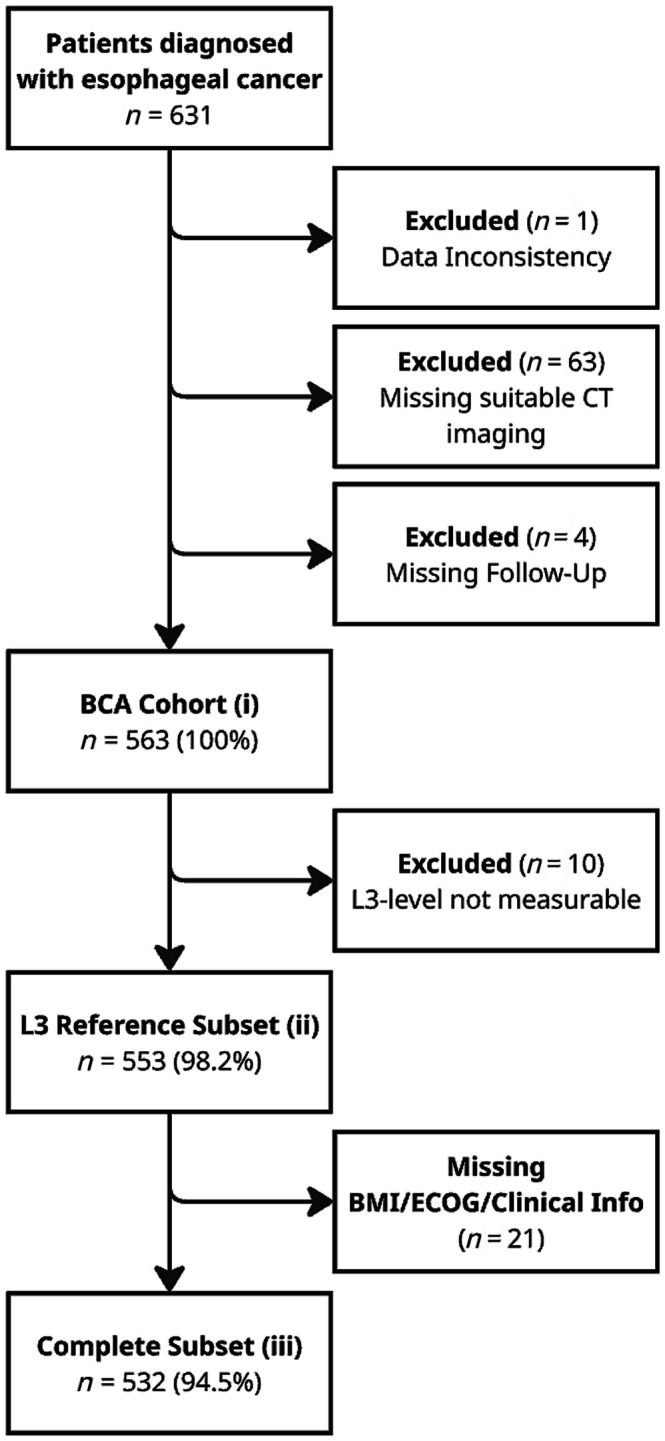
Patient selection flowchart. Of 631 patients diagnosed with oesophageal cancer at our institution (January 2011–December 2024), 1 was excluded for data inconsistency; 63 were excluded for absence of a suitable staging CT (complete abdominal coverage, reconstructed slice thickness ≤ 5 mm and acquired within ±60 days of histological diagnosis); and 4 were excluded for missing follow‐up, leaving 563 patients constituting the (i) BCA cohort. Within this cohort, 10 patients were excluded for unmeasurable L3 vertebral level, yielding the (ii) L3 reference subset (*n* = 553, 98.2%) used for the L3‐derived indices. A further 21 patients were excluded for missing BMI, ECOG performance status or follow‐up time, defining the (iii) complete case analytic cohort (*n* = 532, 94.5%) used for the fully adjusted multivariable Cox models and Δ*C* index analyses.

The patient selection process is summarized in Figure 1.

### 2.2 Patient Characteristics

Clinical variables were extracted from electronic medical records, including demographics (age, sex and date of birth), tumour characteristics (TNM staging according to cTNM classification and metastatic status), treatment information (surgery, chemotherapy and radiation therapy details), ECOG performance status (0–4 scale) and survival endpoints (dates of diagnosis and death and last contact).

The primary endpoint was OS, defined as time from diagnosis to death from any cause. Patients alive at last follow‐up were censored at that date.

### 2.3 AI‐Based 3D BCA Parameters

Automated tissue segmentation was performed using ‘BOA: Body and Organ Analysis’ [23, 24, 25] on the RACOON (Radiological Cooperative Network) infrastructure to quantify tissue volumes in millilitres at the abdominal cavity level. The following compartments were measured: muscle tissue, bone tissue, IMAT, VAT, SAT and total adipose tissue (TAT). Three normalized body composition indices were calculated from these measurements, following the volumetric BCA framework described in detail before [28, 29].

#### 2.3.1 Sarcopenia Index (SI)

Defined as the ratio of muscle volume to bone volume (SI = Muscle/Bone). Bone volume is used as an internal, CT‐intrinsic proxy of body frame size, analogous in purpose to the height^2^ denominator of L3 skeletal muscle index (L3 SMI) but obtained from the same volumetric segmentation rather than from clinical records. This avoids dependence on potentially missing or imprecisely recorded height, and because adult skeletal volume is comparatively stable on the timescale of an oncologic disease course, the denominator does not co‐vary with the disease‐ or treatment‐related changes in soft tissue that the numerator is intended to capture. The resulting ratio reflects muscle mass relative to skeletal frame.

#### 2.3.2 Myosteatotic Fat Index (MFI)

Calculated as MFI = (IMAT/TAT) × 100/5. The (IMAT/TAT) × 100 term expresses myosteatosis as the percentage of TAT that has infiltrated skeletal muscle, normalizing intramuscular fat to each patient's overall adiposity rather than to an absolute volume. Division by 5 is a fixed rescaling so that the hazard ratio reported per one‐unit increase corresponds to a five‐percentage‐point change in IMAT/TAT, an increment that is interpretable on the scale of observed between‐patient variation.

#### 2.3.3 Abdominal Fat Index (AFI)

Defined as AFI = VAT/SAT, representing the ratio of visceral to subcutaneous adiposity, a fat‐distribution rather than a fat‐quantity marker.

We retain the term ‘sarcopenia index’ from the predecessor volumetric BCA studies [28, 29] for cross‐study comparability; together with the MFI and the AFI, it should be interpreted as a CT‐derived imaging surrogate of muscle quantity and quality (and, for AFI, fat distribution), not as a clinical diagnosis of sarcopenia per EWGSOP2 [7] or AWGS 2019 [8], which additionally require muscle strength and/or physical performance assessment and DXA‐ or BIA‐based appendicular lean mass.

### 2.4 L3‐Based Reference BCA Parameters

Established L3‐derived indices were computed for the subset of patients with the L3 vertebra captured on CT (*n* = 553; the L3 reference subset). Each index normalizes the relevant cross‐sectional tissue area at the L3 vertebral level by height squared (cm^2^/m^2^).

#### 2.4.1 L3 Skeletal Muscle Index (L3 SMI)

Muscle cross‐sectional area at L3 normalized by height squared (cm^2^/m^2^); an established CT imaging surrogate of skeletal muscle mass commonly applied in oncologic body composition studies.

#### 2.4.2 L3 Subcutaneous Adipose Tissue Index (L3 SATI)

Subcutaneous adipose tissue cross‐sectional area at L3 normalized by height squared (cm^2^/m^2^) represents the cross‐sectional subcutaneous fat compartment at the L3 landmark.

#### 2.4.3 L3 Visceral Adipose Tissue Index (L3 VATI)

Visceral adipose tissue cross‐sectional area at L3 normalized by height squared (cm^2^/m^2^); represents the intra‐abdominal fat compartment at the L3 landmark.

#### 2.4.4 L3 Intramuscular Adipose Tissue Index (L3 IMATI)

Intramuscular adipose tissue cross‐sectional area at L3 normalized by height squared (cm^2^/m^2^) reflects fatty infiltration within the muscle compartment at the L3 landmark.

### 2.5 Statistical Analysis

**FIGURE 2:**
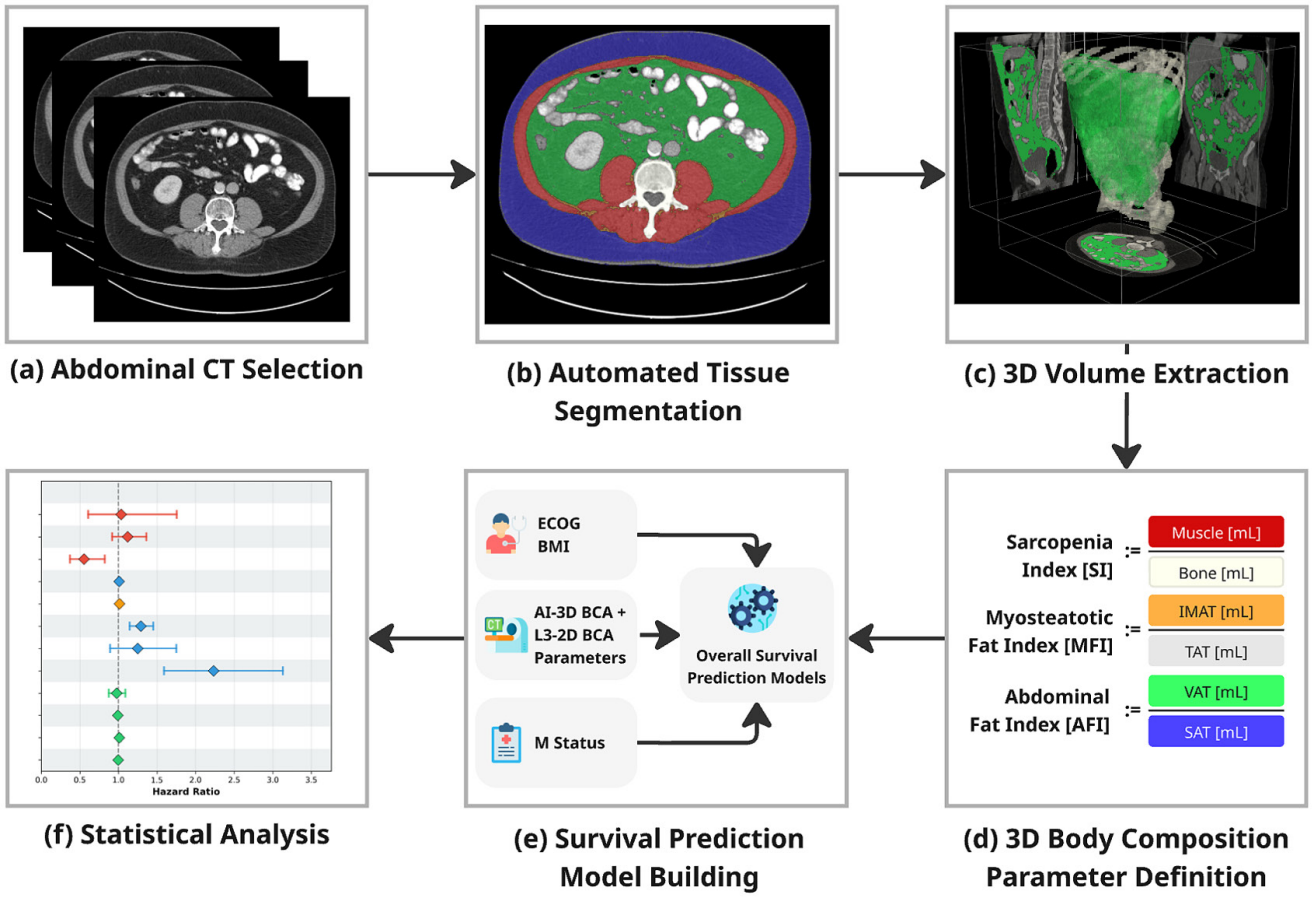
Overview of the AI‐based 3D body composition analysis pipeline for survival prediction. (a) Pretreatment abdominal CT series were selected from patients with histologically confirmed oesophageal cancer (*n* = 563). (b) Automated deep learning segmentation using BOA (Body and Organ Analysis) identified five tissue compartments: muscle (red), bone (white), subcutaneous adipose tissue (SAT, blue), visceral adipose tissue (VAT, green) and intramuscular adipose tissue (IMAT, orange). (c) Tissue volumes were extracted across the entire abdominal cavity. The example image shows the visceral adipose tissue. (d) Three volumetric body composition indices were calculated: sarcopenia index (SI), myosteatotic fat index (MFI) and abdominal fat index (AFI), alongside conventional BMI and L3‐based reference parameters. (e) Overall survival prediction models integrate AI‐derived 3D body composition parameters (AI‐3D BCA) alongside conventional L3‐level 2D measurements (L3‐2D BCA), clinical performance status (ECOG), body mass index (BMI) and metastatic status (M status). (f) Subgroup analyses were performed to assess model performance stratified by sex and metastatic status.

Figure 2 displays an overview of the complete study process. Statistical analyses were performed using Python 3.13.2 with pandas 2.3.3, lifelines 0.30.0, matplotlib 3.10.7 and seaborn 0.13.2.

Distribution normality was assessed by the Shapiro–Wilk test. Normally distributed variables were reported as mean ± standard deviation and compared between sexes using Welch's *t*‐test with Cohen's d effect size; non‐normally distributed variables were reported as median (interquartile range) and compared using the Mann–Whitney U test with rank‐biserial correlation effect size. Follow‐up time was calculated in months from diagnosis to death or last contact.

Kaplan–Meier survival curves were constructed stratified by sex and metastasis (M) status. For visualization, continuous body composition indices were dichotomized at sex‐ and M status–specific median values into high (≥ median) and low (< median) groups, mirroring the approach used in prior volumetric BCA studies in lung cancer [28] and melanoma [29] for cross‐entity comparability. A log‐rank test compared survival between groups, with a minimum group size requirement of five patients. All inferential analyses of SI, MFI and AFI used the indices in continuous form (Cox regression, below); no data‐driven cutpoint search was performed, and no clinical threshold is proposed.

Cox proportional hazards models assessed the independent prognostic value of BCA parameters while controlling for potential confounders. Each BCA parameter was tested in separate models to avoid multicollinearity and combined in a full model. The fully adjusted models included age, sex, metastatic status, ECOG performance status, BMI and L3‐derived indices (L3 SMI, L3 SATI, L3 VATI and L3 IMATI) as covariates (*n* = 532).

Incremental discrimination of each volumetric index beyond the conventional reference parameters was quantified on the primary adjusted Cox model (age, sex, M status, ECOG, BMI, four L3 indices; *n* = 532) by Harrell's *C* index, the change in *C* index (Δ*C*) and a likelihood ratio test, with 95% CIs from a paired percentile bootstrap (*B* = 500) evaluated on the original sample (Table S7).

Variables were coded as follows: sex (male = 1, female = 0), M status (M1 = 1, M0 = 0), ECOG (0–4), with age, BMI and all body composition parameters as continuous variables. Complete case analysis was applied for each model. Models with fewer than 10 patients were excluded. The proportional hazards assumption was tested for the primary adjusted Cox models (age, sex, M status, ECOG, BMI and four L3 indices + marker) and for the combined fully adjusted model via Schoenfeld residual tests with rank time transformation (Table S9); collinearity was assessed by the Pearson correlation matrix of the continuous predictors and by variance inflation factors (VIFs) computed on the design matrix of each Cox fit (Table S10).

The 14‐year inclusion window spans evolving perioperative practice, of which the institutional transition to robot‐assisted minimally invasive esophagectomy (RAMIE) in mid‐2019 was the single most consequential change in surgical management; we used 1 July 2019 as the cutpoint defining a pre‐RAMIE and a RAMIE era. To probe whether any residual temporal effect confounded the BCA estimates, three sensitivity specifications were added to the primary adjusted Cox model (age, sex, M status and ECOG + marker): (i) a binary pre‐RAMIE vs. RAMIE indicator, (ii) stratification of the baseline hazard on this indicator and (iii) diagnosis date as a continuous covariate (years since 1 January 2011), which avoids any cutpoint assumption and captures monotone calendar time trends not aligned with the RAMIE transition. Era‐stratified Kaplan–Meier estimates with 1‐, 3‐ and 5‐year survival (Table S2 and Figure S2), log‐rank tests (Table S3), the case mix comparison across eras (Table S4), the three Cox sensitivity specifications (Table S5), the calendar‐time trend (Table S6) and the diagnosis date histogram with the 1 July 2019 cut‐off (Figure S1) are reported in the Supporting Information. Statistical significance was defined as *p* < 0.05.

## 3 Results

### 3.1 Patient Characteristics

The final BCA cohort comprised 563 patients with oesophageal cancer. The median age at diagnosis was 65.4 years (IQR: 58.8–71.3), with male patients representing 80.3% (*n* = 452) of the cohort. Female patients were by median older at diagnosis (67.3 vs. 64.2 years). M1 was present in 10.1% (*n* = 57) of patients at diagnosis, with a similar distribution between sexes (10.0% male vs. 10.8% female). Surgery was performed in 68.7% (*n* = 387) of patients. ECOG performance status was available for 547 patients (97.2%), with ECOG 0: *n* = 196 (35.8%), 1: *n* = 250 (45.7%), 2: *n* = 66 (12.1%), 3: *n* = 30 (5.5%), 4: *n* = 5 (0.9%). Detailed characteristics can be found in Table 1.

**TABLE 1:** Patient baseline characteristics and perioperative findings. *n* = number of patients, IQR = interquartile range, BMI = body mass index (kg/m^2^), L3 SMI = L3 skeletal muscle index (cm^2^/m^2^), L3 SATI = L3 subcutaneous adipose tissue index (cm^2^/m^2^), L3 VATI = L3 visceral adipose tissue index (cm^2^/m^2^), L3 IMATI = L3 intramuscular adipose tissue index (cm^2^/m^2^), ECOG = Eastern Cooperative Oncology Group performance status and STTH = short‐term targeted therapy; *p* values represent comparisons between males and females at M0 and M1 respectively, using Welch's *t*‐test for sarcopenia index and Mann–Whitney *U* test for all other variables.

Variable	Male (M0)	Female (M0)	*p* (M0)	Male (M1)	Female (M1)	*p* (M1)
Count	*n* = 407	*n* = 99		*n* = 45	*n* = 12	
Age (median, IQR)	64.0 (58.0–70.0)	67.0 (61.0–74.0)	0.008	64.0 (59.0–69.0)	66.0 (61.5–70.0)	0.475
BMI (median, IQR)	25.9 (23.1–29.5)	24.0 (20.4–29.4)	0.002	26.5 (23.0–30.4)	27.1 (24.6–28.4)	0.880
L3 SMI (median, IQR)	14.4 (5.5–21.9)	7.2 (3.8–17.7)	< 0.001	6.5 (5.0–21.2)	4.8 (4.1–10.8)	0.117
L3 SATI (median, IQR)	12.7 (6.0–23.9)	12.3 (7.1–24.2)	0.890	12.7 (5.5–21.0)	13.3 (8.4–27.5)	0.238
L3 VATI (median, IQR)	13.6 (7.2–27.3)	7.0 (3.0–13.8)	< 0.001	10.6 (5.1–16.4)	9.3 (5.3–12.5)	0.591
L3 IMATI (median, IQR)	1.9 (1.0–3.3)	1.5 (1.0–3.2)	0.560	1.6 (0.7–3.0)	1.5 (1.3–2.9)	0.637
BCA parameters						
Sarcopenia index (median, IQR)	2.5 (2.3–2.8)	2.2 (1.9–2.5)	< 0.001	2.5 (2.2–2.8)	2.4 (1.9–2.5)	0.110
Myosteatotic fat index (median, IQR)	2.1 (1.7–2.6)	2.1 (1.8–2.6)	0.818	2.1 (1.8–2.8)	2.0 (1.5–2.6)	0.286
Abdominal fat index (median, IQR)	0.7 (0.6–1.0)	0.4 (0.3–0.5)	< 0.001	0.6 (0.5–0.8)	0.3 (0.3–0.4)	< 0.001
ECOG score						
0	152 (37.3%)	33 (33.3%)		7 (15.6%)	4 (33.3%)	
1	189 (46.4%)	37 (37.4%)		22 (48.9%)	2 (16.7%)	
2	41 (10.1%)	17 (17.2%)		4 (8.9%)	4 (33.3%)	
3	15 (3.7%)	6 (6.1%)		8 (17.8%)	1 (8.3%)	
4	2 (0.5%)	3 (3.0%)		0 (0.0%)	0 (0.0%)	
Preoperative findings						
Histology						
Adenocarcinoma	275 (67.6%)	40 (40.4%)		27 (60.0%)	9 (75.0%)	
Squamous cell carcinoma	103 (25.3%)	49 (49.5%)		16 (35.6%)	1 (8.3%)	
Others	29 (7.1%)	10 (10.1%)		2 (4.4%)	2 (16.7%)	
Grading						
G0	0 (0.0%)	1 (1.0%)		0 (0.0%)	0 (0.0%)	
G1	32 (7.9%)	11 (11.1%)		2 (4.4%)	2 (16.7%)	
G2	195 (47.9%)	47 (47.5%)		17 (37.8%)	5 (41.7%)	
G3	145 (35.6%)	32 (32.3%)		17 (37.8%)	1 (8.3%)	
G4	4 (1.0%)	2 (2.0%)		0 (0.0%)	1 (8.3%)	
Gx	31 (7.6%)	6 (6.1%)		9 (20.0%)	3 (25.0%)	
cT						
cT1	12 (2.9%)	7 (7.1%)		0 (0.0%)	0 (0.0%)	
cT2	84 (20.6%)	21 (21.2%)		4 (8.9%)	0 (0.0%)	
cT3	214 (52.6%)	46 (46.5%)		24 (53.3%)	8 (66.7%)	
cT4	14 (3.4%)	7 (7.1%)		0 (0.0%)	2 (16.7%)	
cTx	83 (20.4%)	18 (18.2%)		17 (37.8%)	2 (16.7%)	
cN						
cN0	63 (15.5%)	20 (20.2%)		2 (4.4%)	0 (0.0%)	
cN1	230 (56.5%)	54 (54.5%)		19 (42.2%)	8 (66.7%)	
cN2	29 (7.1%)	7 (7.1%)		3 (6.7%)	1 (8.3%)	
cN3	9 (2.2%)	0 (0.0%)		4 (8.9%)	1 (8.3%)	
cNx	76 (18.7%)	18 (18.2%)		17 (37.8%)	2 (16.7%)	
Therapy and outcome						
Neoadjuvant therapy						
None	209 (51.4%)	57 (57.6%)		38 (84.4%)	11 (91.7%)	
Chemotherapy	17 (4.2%)	3 (3.0%)		2 (4.4%)	0 (0.0%)	
Radiochemotherapy	179 (44.0%)	37 (37.4%)		4 (8.9%)	1 (8.3%)	
STTH	1 (0.2%)	2 (2.0%)		1 (2.2%)	0 (0.0%)	
Immunotherapy	1 (0.2%)	0 (0.0%)		1 (2.2%)	1 (8.3%)	
Surgery						
Yes	311 (76.4%)	64 (64.6%)		9 (20.0%)	3 (25.0%)	
No	96 (23.6%)	35 (35.4%)		36 (80.0%)	9 (75.0%)	
Residual tumour						
R0	298 (73.2%)	60 (60.6%)		7 (15.6%)	3 (25.0%)	
R1	25 (6.1%)	5 (5.1%)		2 (4.4%)	0 (0.0%)	
Missing	84 (20.6%)	34 (34.3%)		36 (80.0%)	9 (75.0%)	

### 3.2 AI‐Based 3D BCA Parameters

**FIGURE 3:**
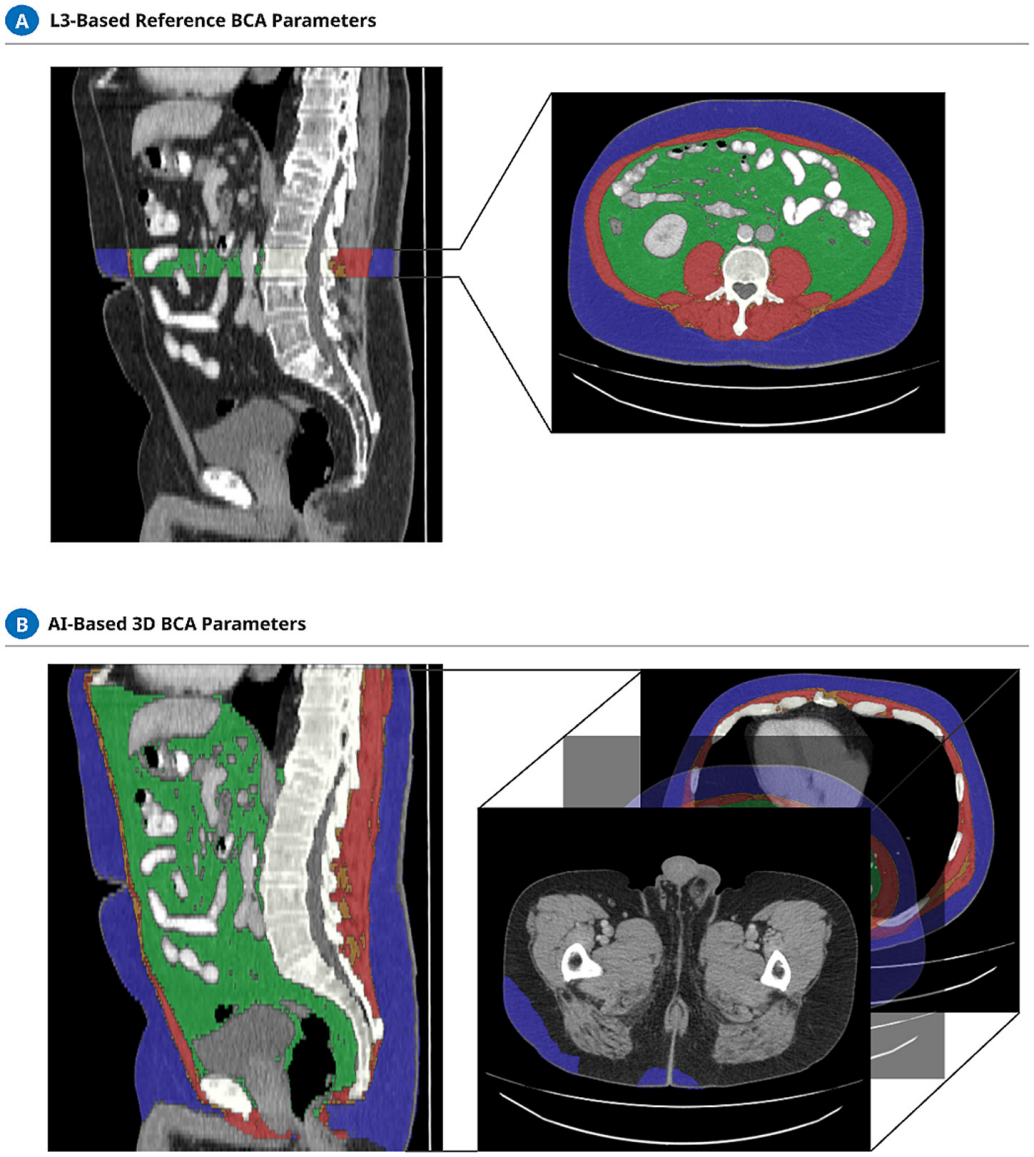
Comparison of conventional L3‐based and AI‐based volumetric body composition analysis. (a) L3‐based reference approach: sagittal CT reconstruction (left) showing the L3 vertebral level used for single‐slice tissue quantification, with corresponding axial segmentation at L3 (right). (b) AI‐based volumetric approach: sagittal view (left) and axial stack (right) demonstrating automated segmentation across the entire abdominal cavity. Tissue compartments are colour‐coded: muscle (red), subcutaneous adipose tissue (blue), visceral adipose tissue (green), intramuscular adipose tissue (orange) and bone (white). The volumetric approach captures total tissue volumes rather than cross‐sectional areas at a single anatomical landmark, providing a more comprehensive assessment of body composition.

Automated segmentation successfully quantified body composition in all 563 patients of the BCA cohort (Figure 3).

#### 3.2.1 Sarcopenia Index (SI)

Distribution testing revealed that the SI followed a normal distribution across all groups (Shapiro–Wilk *p* = 0.409). The SI had a mean of 2.48 ± 0.44, with significantly higher values in male compared with female patients (2.54 ± 0.41 vs. 2.24 ± 0.45, *p* < 0.001, Cohen's *d* = 0.71).

#### 3.2.2 Myosteatotic Fat Index (MFI)

Distribution testing revealed that the MFI showed non‐normal distributions (both *p* < 0.001). MFI had a median of 2.12 (IQR: 1.74–2.60), with no significant sex difference (male: 2.11 [1.74–2.59] vs. female: 2.14 [1.75–2.60], *p* = 0.900, rank‐biserial *r* = −0.008).

#### 3.2.3 Abdominal Fat Index (AFI)

Distribution testing revealed that the AFI showed non‐normal distributions (both *p* < 0.001). The AFI had a median of 0.65 (IQR: 0.46–0.88) and demonstrated marked sex‐specific differences, with male patients showing double the median ratio compared with females (0.72 [0.56–0.95] vs. 0.36 [0.29–0.46], *p* < 0.001, rank‐biserial *r* = −0.77).

#### 3.2.4 3D BCA Parameters Stratified by Metastatic Status

When stratified by metastatic status, significant differences in BCA parameters were observed within the M0 subgroup between sexes for SI (*p* < 0.001) and AFI (*p* < 0.001) but not for MFI (*p* = 0.818). In the M1 subgroup, only the AFI showed significant sex differences (*p* < 0.001), whereas SI (*p* = 0.103) and MFI (*p* = 0.286) did not differ significantly between sexes.

### 3.3 L3‐Based Reference BCA Parameters

L3‐level reference parameters were available for 553 of 563 patients (98.2%), with BMI data available for 547 patients (97.2%). All reference parameters exhibited non‐normal distributions across all subgroups (Shapiro–Wilk *p* < 0.05). The overall cohort demonstrated a median BMI of 25.6 kg/m^2^ (IQR: 22.7–29.6), with male patients showing significantly higher values than female patients (*p* = 0.004, rank‐biserial *r* = −0.18).

L3 SMI demonstrated pronounced sexual dimorphism (male: 14.62 vs. female: 10.08 cm^2^/m^2^, *p* < 0.001, rank‐biserial *r* = −0.32). L3 SATI showed no significant sex difference (*p* = 0.664). L3 VATI was significantly higher in male patients (19.34 vs. 12.26 cm^2^/m^2^, *p* < 0.001, rank‐biserial *r* = −0.32). L3 IMATI showed no significant sex difference (*p* = 0.684).

### 3.4 2D L3‐Based Reference BCA Parameters Stratified by Metastatic Status

Within the M0 subgroup, sex differences remained significant for BMI (*p* = 0.003), L3 SMI (*p* < 0.001) and L3 VATI (*p* < 0.001) but not for L3 SATI (*p* = 0.890) or L3 IMATI (*p* = 0.550). In the M1 subgroup, no significant sex differences were observed for any reference parameter (all *p* > 0.11).

### 3.5 Survival Outcomes

With a median follow‐up of 22.2 months, the median OS for the entire cohort was 28.6 months (95% CI: 23.7–33.3). Among patients with adequate follow‐up, the 1‐year OS rate was 70.7% (390/552, 95% CI: 67.2%–74.8%) and the 5‐year OS rate was 32.8% (112/341, 95% CI: 29.6%–38.3%).

Patients with M1 disease had significantly worse survival compared with M0 patients. The 1‐year survival rate was 46.4% for M1 patients versus 73.4% for M0 patients, whereas 5‐year survival was 10.9% versus 36.3%, respectively. When stratified by both sex and metastatic status, female patients with nonmetastatic disease showed the highest 5‐year survival (40.3%, *n* = 62), whereas female patients with metastatic disease had the lowest (10.0%, *n* = 10).

### 3.6 Kaplan–Meier Analysis

Stratification by sex‐specific median values revealed significant survival differences for multiple body composition parameters.

The SI demonstrated strong prognostic value in both sexes. Male patients with high SI had significantly longer median survival compared with those with low values (33.3 vs. 21.8 months, *p* < 0.001). This association was even more pronounced in female patients (68.8 vs. 16.8 months, *p* < 0.001). When stratified by metastatic status, prognostic significance was maintained in nonmetastatic patients (M0: males 38.8 vs. 24.2 months, *p* < 0.001; females 73.5 vs. 23.3 months, *p* < 0.001) and notably also reached significance in metastatic male patients (M1: 14.6 vs. 7.5 months, *p* = 0.030).

The MFI demonstrated inverse prognostic impact, with high values associated with worse survival in both male (20.9 vs. 34.8 months, *p* < 0.001) and female patients (16.8 vs. 68.5 months, *p* < 0.001). This inverse relationship persisted in nonmetastatic disease (M0: males 22.4 vs. 39.6 months, *p* < 0.001; females 23.7 vs. 73.5 months, *p* < 0.001) but lost significance in metastatic disease (M1: males *p* = 0.057; females *p* = 0.718).

The AFI reached statistical significance only in the female M0 subgroup (31.4 vs. 66.0 months, *p* = 0.035) but not in male patients (*p* = 0.136) or metastatic subgroups.

Among reference parameters, L3 SMI showed significant prognostic value in male patients (38.1 vs. 22.3 months, *p* = 0.003), with this association persisting in the M0 subgroup (40.0 vs. 24.2 months, *p* = 0.026) and M1 subgroup (15.4 vs. 5.98 months, *p* = 0.015). L3 SMI did not reach significance in female patients (*p* = 0.075). BMI showed no significant survival associations in either sex (males *p* = 0.123; females *p* = 0.327) or any metastatic subgroup.

The per‐stratum descriptive comparisons above are based on subgroups of unequal size and event count (Kaplan–Meier analysis cohort): female M0 *n* = 99 (59 deaths), female M1 *n* = 12 (11 deaths), male M0 *n* = 401 (259 deaths), male M1 *n* = 45 (38 deaths) and total *n* = 557 (367 deaths). The descriptive subgroup medians for SI in nonmetastatic female patients (73.5 vs. 23.3 months) and for MFI (73.5 vs. 23.7 months) appear larger than the corresponding contrasts in male patients, but the female metastatic cell is too small to support quantitative subgroup‐level inference. Formal sex‐marker interaction testing, performed on the complete case–adjusted cohort (*n* = 542, 353 deaths; female metastatic *n* = 11, 10 deaths), is reported in Section 3.7.

**FIGURE 4:**
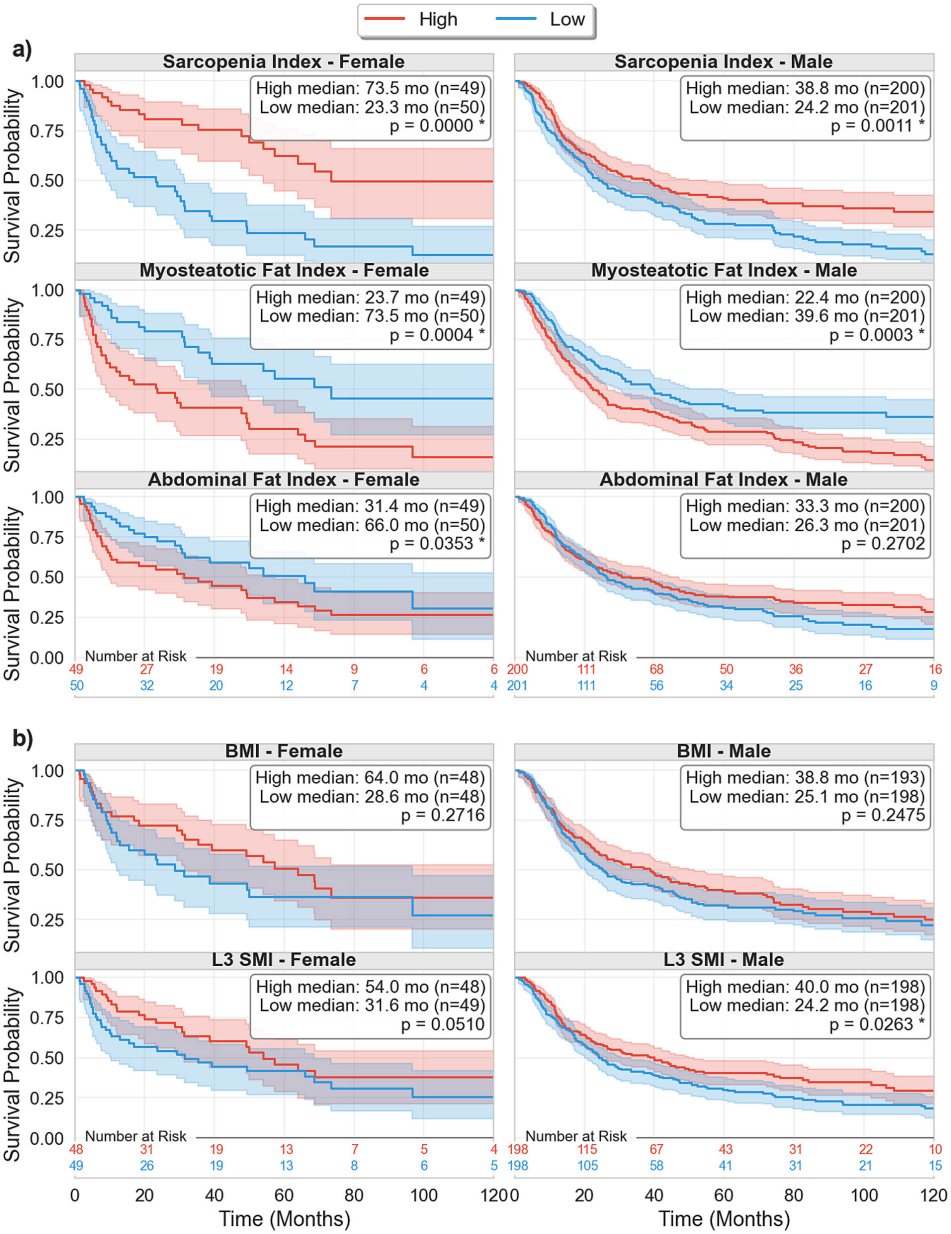
Kaplan–Meier survival curves stratified by body composition markers in M0 patients. (a) Survival analysis for sarcopenia index (SI), myosteatotic fat index (MFI) and abdominal fat index (AFI) stratified by sex. (b) Survival analysis for reference anthropometric parameters (BMI and L3 skeletal muscle index) stratified by sex. Patients were dichotomized into high and low groups based on sex‐specific median values for each marker. Differences between the two groups were measured through log‐rank tests. Coloured areas show the 95% confidence interval.

Figure 4 shows detailed Kaplan Meier plots for M0 patients.

### 3.7 Cox Regression Analysis

In univariate Cox regression, the SI was strongly protective (HR = 0.43, 95% CI: 0.33–0.56, *p* < 0.001), whereas the MFI conferred increased risk (HR = 1.52, 95% CI: 1.33–1.73, *p* < 0.001). The AFI showed no significant association with survival (HR = 1.11, 95% CI: 0.80–1.53, *p* = 0.546).

After adjustment for age, sex and metastatic status (*n* = 557), both SI (HR = 0.40, 95% CI: 0.30–0.54, *p* < 0.001) and MFI (HR = 1.47, 95% CI: 1.28–1.69, *p* < 0.001) remained independent prognostic factors. Age showed significance in models including the MFI (HR = 1.02, 95% CI: 1.00–1.03, *p* = 0.011) or AFI (HR = 1.02, 95% CI: 1.01–1.03, *p* < 0.001) but not in the SI model (HR = 1.01, *p* = 0.341). Male sex was associated with increased risk only in the SI model (HR = 1.38, 95% CI: 1.05–1.80, *p* = 0.021). Metastatic status was consistently prognostic across all models (HR = 2.49–2.51, all *p* < 0.001).

In the subset with available ECOG performance status data (*n* = 542), ECOG was a strong independent predictor across all models (HR = 1.30–1.40, all *p* < 0.001). The SI maintained its protective effect (HR = 0.49, 95% CI: 0.36–0.67, *p* < 0.001), as did the MFI risk association (HR = 1.33, 95% CI: 1.16–1.54, *p* < 0.001). Male sex emerged as a significant risk factor in the SI model (HR = 1.42, 95% CI: 1.08–1.88, *p* = 0.013) but not in the AFI model (HR = 1.25, 95% CI: 0.92–1.71, *p* = 0.161).

When adjusting for Body Mass Index (BMI) (*n* = 541), both SI (HR = 0.42, 95% CI: 0.30–0.57, *p* < 0.001) and MFI (HR = 1.45, 95% CI: 1.25–1.67, *p* < 0.001) retained significance, whereas BMI itself was not independently prognostic in any model (all *p* > 0.05).

Adjustment for L3‐derived body composition indices (*n* = 547) did not attenuate the associations of the BCA parameters. The SI (HR = 0.43, 95% CI: 0.30–0.60, *p* < 0.001) and MFI (HR = 1.44, 95% CI: 1.22–1.70, *p* < 0.001) remained significant, whereas none of the L3 indices (L3 SMI, L3 SATI, L3 VATI and L3 IMATI) achieved independent significance (all *p* > 0.05).

In the fully adjusted models incorporating age, sex, M status, ECOG, BMI and L3 indices (*n* = 532), both SI (HR = 0.50, 95% CI: 0.35–0.72, *p* < 0.001) and MFI (HR = 1.27, 95% CI: 1.06–1.51, *p* = 0.009) maintained independent prognostic value. The AFI remained non‐significant (HR = 1.06, 95% CI: 0.63–1.80, *p* = 0.825).

**FIGURE 5:**
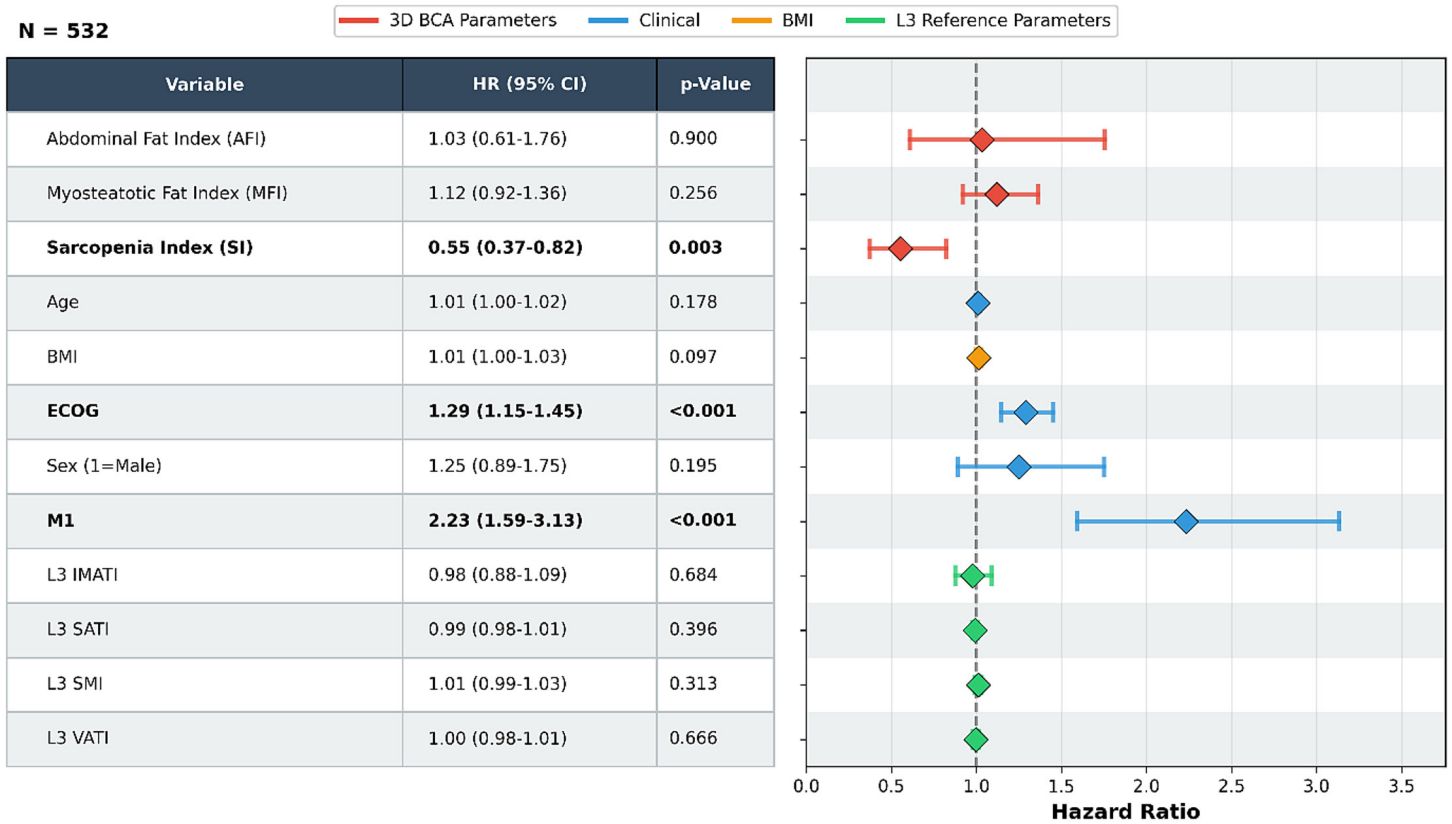
Cox Regression Results for our fully adjusted model. The fully adjusted model included all three volumetric body composition indices (red), clinical covariates (blue: age, sex, metastatic status [M1], ECOG performance status), BMI (orange) and conventional L3‐based reference parameters (green: L3 SMI, L3 SATI, L3 VATI and L3 IMATI). The Sarcopenia Index demonstrated independent protective effects (HR = 0.56, 95% CI: 0.37–0.82, *p* = 0.003), while metastatic status (HR = 2.23, *p* < 0.001) and ECOG performance status (HR = 1.29, *p* < 0.001) remained significant predictors of mortality. Notably, all L3‐derived indices lost statistical significance when included alongside volumetric parameters. The dashed vertical line indicates HR = 1.0 (no effect). Bold text denotes statistically significant associations (*p* < 0.05). *N* = 532 patients with complete data.

In the combined model incorporating all three BCA parameters simultaneously with full covariate adjustment (*n* = 532), only the SI retained independent prognostic significance (HR = 0.56, 95% CI: 0.37–0.82, *p* = 0.003). The MFI (HR = 1.12, 95% CI: 0.92–1.36, *p* = 0.256) and AFI (HR = 1.03, 95% CI: 0.61–1.76, *p* = 0.900) were not independently associated with survival when mutually adjusted. Metastatic status (HR = 2.23, 95% CI: 1.59–3.13, *p* < 0.001) and ECOG performance status (HR = 1.29, 95% CI: 1.15–1.45, *p* < 0.001) remained significant predictors. The results of the full model are shown in Figure 5; the complete multivariable table with hazard ratios, 95% confidence intervals and *p* values for every covariate across all primary, fully adjusted and combined Cox specifications is provided in Table S1.

**FIGURE 6:**
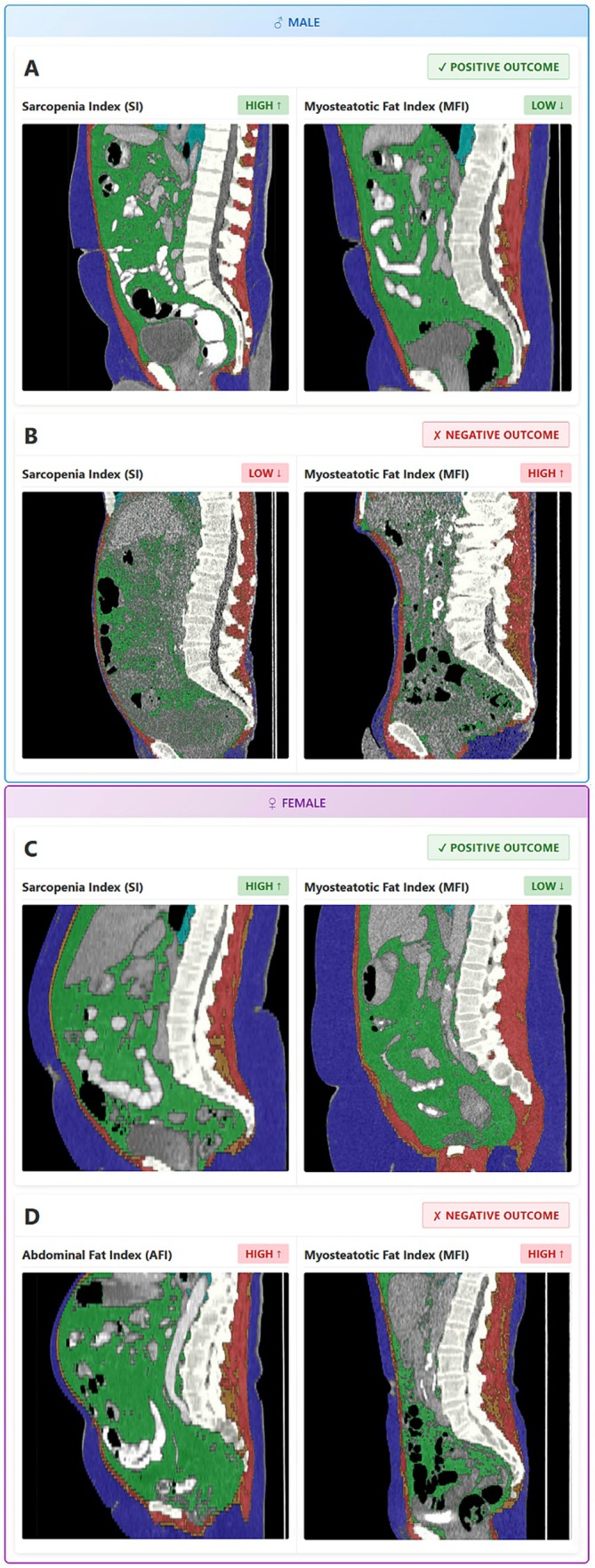
Representative sagittal CT images from the study cohort demonstrating imaging parameters stratified by sex and clinical outcome. Each parameter is measured from a different representative patient. (A) Male patients with positive outcome demonstrating high sarcopenia index (SI) and low myosteatotic fat index (MFI) (SI: 46y AC, T3N1M0, G2, OS 59.3 months, full remission; MFI: 69y adenocarcinoma, T3N2M0, G3, OS 22.8 months, deceased). (B) Male patients with negative outcome demonstrating low sarcopenia index (SI) and high MFI (SI: 58y epithelial carcinoma, T1N0M0, unspecified, OS 1.2 months, deceased; MFI: 67y squamous cell carcinoma, T3N1M0, G2, OS 6.6 months, deceased). (C) Female patients with positive outcome demonstrating high Sarcopenia Index (SI) and low MFI (SI: 56y adenocarcinoma, T2N0M0, G3, OS 172 months, full remission; MFI: 51y adenocarcinoma, T3N1M0, G3, OS 51.3 months, full remission). (D) Female patients with negative outcome demonstrating high abdominal fat index (AFI) and high MFI (AFI: 82y adenocarcinoma, T3N1M0, G3, OS 9.5 months, deceased; MFI: 60y squamous cell carcinoma, T3N0M0, G2, OS 49.2 months, deceased).

Consistent independent predictors across fully adjusted models included metastatic status (HR = 2.23–2.28, all *p* < 0.001) and ECOG performance status (HR = 1.31–1.39, all *p* < 0.001). Male sex did not reach independent significance in any fully adjusted model (all *p* > 0.05). Neither BMI nor any L3 index achieved independent significance in the fully adjusted models (all *p* > 0.05). Figure 6 displays representative examples of the study cohort demonstrating the imaging parameters.

Added to the primary adjusted reference model (age, sex, M status, ECOG, BMI and four L3 indices; *n* = 532, *C* index 0.625), the SI improved discrimination (*C* index 0.641, Δ*C* = +0.015, 95% CI +0.007 to +0.018; LRT *p* < 0.001) and the MFI added a smaller increment (*C* index 0.633, Δ*C* = +0.007, 95% CI 0.000 to +0.011; *p* = 0.011), whereas the AFI did not (Δ*C* = +0.0002, 95% CI −0.009 to +0.001; *p* = 0.83).

Full results across all marker adjustment combinations are reported in Table S7.

OS did not differ between the pre‐RAMIE (*n* = 291) and RAMIE eras (*n* = 267; log‐rank *p* = 0.68; median 28.8 vs. 26.3 months), and calendar time was not independently prognostic in the primary adjusted model (HR per +1 year 1.010, 95% CI 0.982–1.040, *p* = 0.48). Applied to the primary adjusted Cox model (age, sex, M status and ECOG + marker), adding the era indicator, stratifying on it or replacing it with continuous calendar time changed each BCA hazard ratio by less than 2% with no change in significance (Tables S5 and S6).

Formal sex marker interaction tests on the primary adjusted Cox model (age, sex, M status, ECOG + marker; *n* = 542 complete case) confirmed that the SI effect differs by sex (interaction HR for males vs. females 2.23, 95% CI 1.09–4.56; likelihood ratio test *p* = 0.026): the protective association was larger in females (female‐only HR 0.25, 95% CI 0.12–0.49) than in males (male‐only HR 0.60, 95% CI 0.42–0.84). The sex–MFI interaction was borderline (interaction HR 0.72, 95% CI 0.52–1.00; LRT *p* = 0.053; female‐only HR 1.76, 95% CI 1.30–2.38; male‐only HR 1.24, 95% CI 1.05–1.46), directionally consistent with a larger effect in females but not formally significant. The sex‐AFI interaction was not supported (LRT *p* = 0.164); the female‐only estimate is wide and imprecise (HR 3.59, 95% CI 0.77–16.72) and not interpretable as an effect size. Three‐way sex‐M1‐marker interactions were not fitted (see Section 3.5). Subgroup counts and the full interaction results are provided in Table S8.

## 4 Discussion

In this study, we investigated the prognostic impact of AI‐derived volumetric body composition parameters in 563 patients with oesophageal cancer. The study was necessitated by the limited objective measures available for individualized treatment planning in this malignancy, particularly given the well‐recognized but incompletely quantified influence of body composition on perioperative and oncological outcomes. Our results demonstrated that the SI and MFI provide independent prognostic information after full adjustment for age, sex, metastatic status, ECOG performance status, BMI and all four conventional L3‐based indices, with the volumetric markers outperforming and rendering the L3 indices non‐significant. To our knowledge, these findings represent the first comprehensive assessment of automated three‐dimensional body composition parameters in a large, well‐characterized cohort of oesophageal cancer patients, demonstrating that fully automatic CT‐based body composition analysis at staging is feasible and prognostically informative without additional diagnostic burden.

### 4.1 Comparison With Existing Literature

Although CT imaging–defined low muscle mass has been consistently linked to adverse outcomes in oesophageal cancer across multiple meta‐analyses [5, 6, 17], the present study extends this evidence in two further directions. First, three‐dimensional volumetric segmentation outperforms conventional L3‐based indices as an independent survival predictor: a finding with potential clinical implications, given that L3 landmark identification can be complicated by mediastinal tumour extension and post‐treatment anatomical distortion characteristic of oesophageal cancer [22]. Second, the independent association of myosteatosis with mortality, backed by Aleixo et al. [14], addresses a dimension of muscle quality that is systematically underrepresented in oesophageal cancer research yet likely reflects its cachectic and pro‐inflammatory tumour microenvironment. The formal sex‐SI interaction, with a substantially stronger protective association in females than in males, provides a mechanistic candidate explanation for the previously reported female survival advantage in predominantly squamous cell carcinoma [32, 33] and underscores the need for sex‐stratified interpretation in future prognostic algorithms. Our findings also show notable consistency with 3D BCA‐parameters in lung cancer [28] and melanoma [29], supporting a tumour‐agnostic role of body composition as a physiological reserve marker that is particularly consequential in oesophageal cancer given the morbidity of esophagectomy and the intensity of multimodal neoadjuvant regimens [34, 35].

### 4.2 Biological Plausibility and Clinical Implications

The mechanisms linking low muscle mass and myosteatosis to adverse outcomes are particularly relevant to oesophageal cancer, where the catabolic burden of the disease, neoadjuvant chemoradiotherapy and major surgery converge. Skeletal muscle serves as an amino acid reservoir and an endocrine organ secreting myokines that modulate systemic inflammation and tumour immunity [6]; diminished muscular reserve translates directly into reduced treatment tolerance, greater postoperative morbidity and impaired recovery [9, 10, 11]. Myosteatosis reflects systemic metabolic dysregulation with lipotoxic effects impairing insulin signalling and amplifying inflammatory cytokine production [12, 13, 16], a milieu that may accelerate tumour progression and attenuate immune surveillance. The absence of independent prognostic significance for the AFI is consistent with the opposing metabolic roles of visceral versus subcutaneous adipose tissue, with subcutaneous fat paradoxically associated with improved survival [15]. In oesophageal cancer, where guideline‐defined sarcopenia assessment (EWGSOP2 [7], AWGS 2019 [8]) is not part of routine pretreatment workup, CT‐derived volumetric indices may offer a practically accessible surrogate that captures cachexia trajectory, metabolic dysregulation and physiological reserve: domains collectively relevant to chemotherapy tolerance, surgical fitness and post‐esophagectomy recovery [9, 11, 14] without additional examinations. Clinically, a composite body composition score derived from staging CT could complement ECOG status as a fitness‐for‐treatment criterion, for example, to individualize the decision between perioperative FLOT chemotherapy and definitive chemoradiotherapy in borderline surgical candidates, representing an ‘opportunistic’ use of imaging [37, 38] that adds no radiation burden. Such use is hypothesis‐generating rather than established and would require prospective validation before informing treatment decisions. The automated nnU‐Net‐based pipeline (Sørensen‐Dice 0.935) [23, 25] circumvents the disease‐specific anatomical challenge of L3‐slice selection near the thoracoabdominal junction and delivers segmentation within minutes; external multi‐institutional validation of survival discrimination (*p* = 0.044) [28] provides preliminary evidence that the prognostic signal generalizes across heterogeneous CT protocols.

### 4.3 Study Limitations and Strengths

Several limitations warrant acknowledgment and moderate the clinical interpretation of these findings. The retrospective single‐centre design limits generalizability. However, this study represents the largest number of patients in this context. The present results were not externally validated and confirmation in independent oesophageal cancer cohorts is required as the next step before clinical translation. Moreover, despite hierarchical adjustment for age, sex, metastatic status, ECOG performance status, BMI and the four L3 indices, residual confounding from unmeasured factors (e.g., comorbidity burden, nutritional status, smoking and alcohol exposure) remained unconsidered. The incremental gain in discrimination over the established predictors was likewise modest, and calibration and net clinical benefit were not assessed; these factors represent next steps for future clinical translation. The heterogeneous treatment approaches spanning 14 years introduce therapeutic variability, although sensitivity analyses confirmed that the RAMIE era transition (mid‐2019) did not materially alter the BCA hazard ratios and OS did not differ between eras (log‐rank *p* = 0.68). The absence of granular data on specific chemotherapy regimens (e.g., FLOT vs. cisplatin‐based protocols), radiation doses and surgical technique prevents detailed analysis of treatment body composition interactions. Most critically, we lacked longitudinal body composition measurements to capture compositional changes during neoadjuvant therapy, which is a dimension identified as independently informative by Wang et al. [10] and Luo et al. [9] and the most actionable clinical extension of the present findings. Important strengths include the large cohort (*n* = 563) with near‐complete automated segmentation, comprehensive ECOG annotation (97.2%) and rigorous hierarchical model construction with formal sensitivity analyses; the consistent significance of SI and MFI across all model specifications, from simple clinical adjustment through the fully adjusted model including all four L3 indices, provides robust evidence for their independent prognostic value. We deliberately do not propose clinical cut‐offs: Data‐driven thresholds from a single retrospective cohort are prone to Type‐I inflation and prospective derivation in oesophageal cancer–specific external cohorts is required. The small female metastatic subgroup (*n* = 11) precludes precise subgroup effect size estimates; future studies should ensure sufficient female and M1 representation.

### 4.4 Future Directions

Prospective external validation of the SI and MFI is the immediate priority, particularly within dedicated oesophageal cancer cohorts and the neoadjuvant setting, with pre‐specified decision thresholds, calibration and net clinical benefit assessment. Sex‐stratified study designs are indicated by the formally demonstrated sex‐SI interaction. Prehabilitation trials combining nutritional optimization, supervised exercise and protein supplementation represent a therapeutically actionable downstream application: Low SI or high MFI at staging could serve as entry criteria for structured prehabilitation programs preceding esophagectomy or neoadjuvant therapy. Longitudinal body composition assessment during neoadjuvant chemoradiotherapy, to capture therapy‐induced muscle depletion as a dynamic treatment‐response biomarker, and integration with inflammatory, molecular and functional‐imaging biomarkers within multidimensional prognostic models [39] are natural extensions of the present work.

## 5 Conclusion

In conclusion, AI‐derived volumetric body composition parameters, specifically the SI and MFI, provide independent prognostic information in patients with oesophageal cancer beyond established clinical parameters and conventional L3 measurements and modestly improve overall‐survival discrimination. The automated three‐dimensional assessment is extractable from routine staging CT without additional imaging burden, motivating its further development as a candidate component of oncological risk stratification, with the prospect of identifying patients who may benefit from targeted nutritional or muscle‐directed interventions pending confirmation in prospective studies. Given the single‐centre retrospective design and the absence of external validation, the present findings are a solid base for potential future clinical translation.

## Funding

This study was supported by German Netzwerk Universitätsmedizin 3.0, project RACOON (FKZ/grant number: 01KX2524).

## Ethics Statement

The study was approved by the institutional Ethics Commission Westphalia‐Lippe, approval No. 20224‐269‐f‐S and conducted in accordance with the Declaration of Helsinki. Due to the retrospective nature of the study, informed consent was waived by the ethics committee. All data were fully anonymized before being included in the study.

## Conflicts of Interest

The authors declare no conflicts of interest.

## Supporting information


**Table S1:** Complete multivariable Cox regression.
**Table S2:** Era‐stratified overall survival.
**Table S3:** Log‐rank tests, pre‐RAMIE versus RAMIE.
**Table S4:** Case mix across eras.
**Table S5:** Cox sensitivity to era specification.
**Table S6:** Calendar time trend.
**Table S7:** Incremental discrimination (C‐index).
**Table S8:** Sex x marker interactions and subgroup counts.
**Table S9:** Proportional hazards diagnostics.
**Table S10:** Collinearity: correlation and VIF.
**Figure S1:** Diagnosis dates with the RAMIE cutpoint.
**Figure S2:** Era‐stratified Kaplan–Meier curves.

## Data Availability

The data that support the findings of this study are available on request from the corresponding author. The data are not publicly available due to privacy or ethical restrictions.
