# Supplementary material for: Prediction of Overall Survival in Patients With Oesophageal Cancer Using AI‐Based 3D CT Body Composition Analysis
Source: J Cachexia Sarcopenia Muscle. 2026 Aug 11;17(4):e70363. doi: 10.1002/jcsm.70363 (PMC13462676; doi:10.1002/jcsm.70363)
Supplement: Supplementary file 1 [file JCSM-17-e70363-s001.docx]

**Supplementary Material**

*Prediction of overall survival in patients with esophageal cancer using AI-based 3D CT body composition analysis*

# Supplementary Table S1. Complete multivariable Cox regression

| Model | Covariates | Model Context | Variable | HR (95% CI) | p-value | Sig. | N | C-index |
| --- | --- | --- | --- | --- | --- | --- | --- | --- |
| Univariate | None (unadjusted) | Abdominal Fat Index (AFI) | Abdominal Fat Index (AFI) | 1.11 (0.80 to 1.53) | 0.546 |  | 557 | 0.516 |
|  |  | Myosteatotic Fat Index (MFI) | Myosteatotic Fat Index (MFI) | 1.52 (1.33 to 1.73) | <0.001 | *** | 557 | 0.582 |
|  |  | Sarcopenia Index (SI) | Sarcopenia Index (SI) | 0.43 (0.33 to 0.56) | <0.001 | *** | 557 | 0.592 |
| Base Model | Age, Sex, M-Status | Base Model | Age | 1.02 (1.01 to 1.03) | <0.001 | *** | 557 | 0.582 |
|  |  |  | M1 | 2.51 (1.85 to 3.40) | <0.001 | *** | 557 | 0.582 |
|  |  |  | Sex (1=Male) | 1.15 (0.89 to 1.50) | 0.287 |  | 557 | 0.582 |
| Full Model | Age, Sex, M-Status, All BCA markers | Full Model | Abdominal Fat Index (AFI) | 1.08 (0.73 to 1.59) | 0.710 |  | 557 | 0.631 |
|  |  |  | Age | 1.01 (0.99 to 1.02) | 0.330 |  | 557 | 0.631 |
|  |  |  | M1 | 2.51 (1.85 to 3.42) | <0.001 | *** | 557 | 0.631 |
|  |  |  | Myosteatotic Fat Index (MFI) | 1.20 (1.00 to 1.43) | 0.046 | * | 557 | 0.631 |
|  |  |  | Sarcopenia Index (SI) | 0.50 (0.35 to 0.72) | <0.001 | *** | 557 | 0.631 |
|  |  |  | Sex (1=Male) | 1.25 (0.91 to 1.71) | 0.160 |  | 557 | 0.631 |
| Full + BMI | Age, Sex, M-Status, All BCA markers, BMI | Full Model Bmi | Abdominal Fat Index (AFI) | 1.09 (0.74 to 1.63) | 0.656 |  | 541 | 0.628 |
|  |  |  | Age | 1.01 (0.99 to 1.02) | 0.333 |  | 541 | 0.628 |
|  |  |  | BMI | 1.00 (0.99 to 1.02) | 0.752 |  | 541 | 0.628 |
|  |  |  | M1 | 2.43 (1.76 to 3.35) | <0.001 | *** | 541 | 0.628 |
|  |  |  | Myosteatotic Fat Index (MFI) | 1.21 (1.01 to 1.44) | 0.042 | * | 541 | 0.628 |
|  |  |  | Sarcopenia Index (SI) | 0.52 (0.35 to 0.76) | <0.001 | *** | 541 | 0.628 |
|  |  |  | Sex (1=Male) | 1.21 (0.88 to 1.67) | 0.246 |  | 541 | 0.628 |
| Full + L3 | Age, Sex, M-Status, All BCA markers, L3 indices | Full Model L3 | Abdominal Fat Index (AFI) | 1.09 (0.65 to 1.82) | 0.753 |  | 547 | 0.627 |
|  |  |  | Age | 1.01 (1.00 to 1.02) | 0.217 |  | 547 | 0.627 |
|  |  |  | L3 IMATI | 0.94 (0.85 to 1.04) | 0.238 |  | 547 | 0.627 |
|  |  |  | L3 SATI | 1.00 (0.99 to 1.02) | 0.770 |  | 547 | 0.627 |
|  |  |  | L3 SMI | 1.00 (0.98 to 1.02) | 0.886 |  | 547 | 0.627 |
|  |  |  | L3 VATI | 1.00 (0.98 to 1.02) | 0.989 |  | 547 | 0.627 |
|  |  |  | M1 | 2.43 (1.76 to 3.36) | <0.001 | *** | 547 | 0.627 |
|  |  |  | Myosteatotic Fat Index (MFI) | 1.22 (1.01 to 1.48) | 0.038 | * | 547 | 0.627 |
|  |  |  | Sarcopenia Index (SI) | 0.51 (0.35 to 0.75) | <0.001 | *** | 547 | 0.627 |
|  |  |  | Sex (1=Male) | 1.27 (0.92 to 1.77) | 0.147 |  | 547 | 0.627 |
| Base + ECOG | Age, Sex, M-Status, ECOG | Base Model Ecog | Age | 1.02 (1.01 to 1.03) | 0.001 | ** | 542 | 0.62 |
|  |  |  | ECOG | 1.40 (1.25 to 1.56) | <0.001 | *** | 542 | 0.62 |
|  |  |  | M1 | 2.25 (1.63 to 3.11) | <0.001 | *** | 542 | 0.62 |
|  |  |  | Sex (1=Male) | 1.26 (0.96 to 1.65) | 0.098 |  | 542 | 0.62 |
| Full + ECOG | Age, Sex, M-Status, ECOG, All BCA markers | Full Model Ecog | Abdominal Fat Index (AFI) | 1.02 (0.68 to 1.51) | 0.941 |  | 542 | 0.642 |
|  |  |  | Age | 1.01 (1.00 to 1.02) | 0.204 |  | 542 | 0.642 |
|  |  |  | ECOG | 1.28 (1.15 to 1.44) | <0.001 | *** | 542 | 0.642 |
|  |  |  | M1 | 2.32 (1.68 to 3.20) | <0.001 | *** | 542 | 0.642 |
|  |  |  | Myosteatotic Fat Index (MFI) | 1.14 (0.96 to 1.36) | 0.129 |  | 542 | 0.642 |
|  |  |  | Sarcopenia Index (SI) | 0.58 (0.40 to 0.83) | 0.003 | ** | 542 | 0.642 |
|  |  |  | Sex (1=Male) | 1.34 (0.97 to 1.84) | 0.077 |  | 542 | 0.642 |
| Full + ECOG + BMI | Age, Sex, M-Status, ECOG, All BCA markers, BMI | Full Model Ecog Bmi | Abdominal Fat Index (AFI) | 1.05 (0.70 to 1.56) | 0.829 |  | 532 | 0.64 |
|  |  |  | Age | 1.01 (1.00 to 1.02) | 0.216 |  | 532 | 0.64 |
|  |  |  | BMI | 1.00 (0.99 to 1.02) | 0.793 |  | 532 | 0.64 |
|  |  |  | ECOG | 1.28 (1.14 to 1.44) | <0.001 | *** | 532 | 0.64 |
|  |  |  | M1 | 2.28 (1.64 to 3.18) | <0.001 | *** | 532 | 0.64 |
|  |  |  | Myosteatotic Fat Index (MFI) | 1.15 (0.96 to 1.37) | 0.132 |  | 532 | 0.64 |
|  |  |  | Sarcopenia Index (SI) | 0.58 (0.40 to 0.85) | 0.006 | ** | 532 | 0.64 |
|  |  |  | Sex (1=Male) | 1.28 (0.92 to 1.78) | 0.138 |  | 532 | 0.64 |
| Full + ECOG + L3 | Age, Sex, M-Status, ECOG, All BCA markers, L3 indices | Full Model Ecog L3 | Abdominal Fat Index (AFI) | 0.96 (0.57 to 1.62) | 0.881 |  | 538 | 0.642 |
|  |  |  | Age | 1.01 (1.00 to 1.02) | 0.153 |  | 538 | 0.642 |
|  |  |  | ECOG | 1.29 (1.14 to 1.45) | <0.001 | *** | 538 | 0.642 |
|  |  |  | L3 IMATI | 0.97 (0.87 to 1.08) | 0.571 |  | 538 | 0.642 |
|  |  |  | L3 SATI | 1.00 (0.98 to 1.01) | 0.601 |  | 538 | 0.642 |
|  |  |  | L3 SMI | 1.01 (0.99 to 1.02) | 0.611 |  | 538 | 0.642 |
|  |  |  | L3 VATI | 1.00 (0.98 to 1.02) | 0.997 |  | 538 | 0.642 |
|  |  |  | M1 | 2.27 (1.63 to 3.17) | <0.001 | *** | 538 | 0.642 |
|  |  |  | Myosteatotic Fat Index (MFI) | 1.13 (0.93 to 1.37) | 0.214 |  | 538 | 0.642 |
|  |  |  | Sarcopenia Index (SI) | 0.58 (0.39 to 0.85) | 0.005 | ** | 538 | 0.642 |
|  |  |  | Sex (1=Male) | 1.34 (0.96 to 1.86) | 0.089 |  | 538 | 0.642 |
| Full + ECOG + BMI + L3 | Age, Sex, M-Status, ECOG, All BCA markers, BMI, L3 indices | Full Model Ecog Bmi L3 | Abdominal Fat Index (AFI) | 1.03 (0.61 to 1.76) | 0.900 |  | 532 | 0.641 |
|  |  |  | Age | 1.01 (1.00 to 1.02) | 0.178 |  | 532 | 0.641 |
|  |  |  | BMI | 1.01 (1.00 to 1.03) | 0.097 |  | 532 | 0.641 |
|  |  |  | ECOG | 1.29 (1.15 to 1.45) | <0.001 | *** | 532 | 0.641 |
|  |  |  | L3 IMATI | 0.98 (0.88 to 1.09) | 0.684 |  | 532 | 0.641 |
|  |  |  | L3 SATI | 0.99 (0.98 to 1.01) | 0.396 |  | 532 | 0.641 |
|  |  |  | L3 SMI | 1.01 (0.99 to 1.03) | 0.313 |  | 532 | 0.641 |
|  |  |  | L3 VATI | 1.00 (0.98 to 1.01) | 0.666 |  | 532 | 0.641 |
|  |  |  | M1 | 2.23 (1.59 to 3.13) | <0.001 | *** | 532 | 0.641 |
|  |  |  | Myosteatotic Fat Index (MFI) | 1.12 (0.92 to 1.36) | 0.256 |  | 532 | 0.641 |
|  |  |  | Sarcopenia Index (SI) | 0.56 (0.37 to 0.82) | 0.003 | ** | 532 | 0.641 |
|  |  |  | Sex (1=Male) | 1.25 (0.89 to 1.75) | 0.195 |  | 532 | 0.641 |
| Clinical + Single BCA | Age, Sex, M-Status, Single BCA marker | Abdominal Fat Index (AFI) | Abdominal Fat Index (AFI) | 1.02 (0.69 to 1.51) | 0.924 |  | 557 | 0.582 |
|  |  |  | Age | 1.02 (1.01 to 1.03) | <0.001 | *** | 557 | 0.582 |
|  |  |  | M1 | 2.51 (1.85 to 3.42) | <0.001 | *** | 557 | 0.582 |
|  |  |  | Sex (1=Male) | 1.15 (0.84 to 1.55) | 0.386 |  | 557 | 0.582 |
|  |  | Myosteatotic Fat Index (MFI) | Age | 1.02 (1.00 to 1.03) | 0.011 | * | 557 | 0.621 |
|  |  |  | M1 | 2.49 (1.84 to 3.38) | <0.001 | *** | 557 | 0.621 |
|  |  |  | Myosteatotic Fat Index (MFI) | 1.47 (1.28 to 1.69) | <0.001 | *** | 557 | 0.621 |
|  |  |  | Sex (1=Male) | 1.09 (0.84 to 1.42) | 0.504 |  | 557 | 0.621 |
|  |  | Sarcopenia Index (SI) | Age | 1.01 (0.99 to 1.02) | 0.341 |  | 557 | 0.625 |
|  |  |  | M1 | 2.49 (1.83 to 3.37) | <0.001 | *** | 557 | 0.625 |
|  |  |  | Sarcopenia Index (SI) | 0.40 (0.30 to 0.54) | <0.001 | *** | 557 | 0.625 |
|  |  |  | Sex (1=Male) | 1.38 (1.05 to 1.80) | 0.021 | * | 557 | 0.625 |
| Clinical + BMI + Single BCA | Age, Sex, M-Status, BMI, Single BCA marker | Abdominal Fat Index (AFI) | Abdominal Fat Index (AFI) | 1.01 (0.68 to 1.50) | 0.970 |  | 541 | 0.592 |
|  |  |  | Age | 1.02 (1.01 to 1.03) | <0.001 | *** | 541 | 0.592 |
|  |  |  | BMI | 0.98 (0.96 to 1.00) | 0.074 |  | 541 | 0.592 |
|  |  |  | M1 | 2.54 (1.84 to 3.49) | <0.001 | *** | 541 | 0.592 |
|  |  |  | Sex (1=Male) | 1.17 (0.86 to 1.61) | 0.323 |  | 541 | 0.592 |
|  |  | Myosteatotic Fat Index (MFI) | Age | 1.02 (1.00 to 1.03) | 0.011 | * | 541 | 0.614 |
|  |  |  | BMI | 1.00 (0.98 to 1.01) | 0.556 |  | 541 | 0.614 |
|  |  |  | M1 | 2.46 (1.79 to 3.38) | <0.001 | *** | 541 | 0.614 |
|  |  |  | Myosteatotic Fat Index (MFI) | 1.45 (1.25 to 1.67) | <0.001 | *** | 541 | 0.614 |
|  |  |  | Sex (1=Male) | 1.09 (0.83 to 1.44) | 0.523 |  | 541 | 0.614 |
|  |  | Sarcopenia Index (SI) | Age | 1.01 (0.99 to 1.02) | 0.339 |  | 541 | 0.621 |
|  |  |  | BMI | 1.00 (0.98 to 1.02) | 0.885 |  | 541 | 0.621 |
|  |  |  | M1 | 2.41 (1.75 to 3.31) | <0.001 | *** | 541 | 0.621 |
|  |  |  | Sarcopenia Index (SI) | 0.42 (0.30 to 0.57) | <0.001 | *** | 541 | 0.621 |
|  |  |  | Sex (1=Male) | 1.35 (1.02 to 1.78) | 0.033 | * | 541 | 0.621 |
| Clinical + L3 + Single BCA | Age, Sex, M-Status, L3 indices, Single BCA marker | Abdominal Fat Index (AFI) | Abdominal Fat Index (AFI) | 1.09 (0.65 to 1.82) | 0.753 |  | 547 | 0.595 |
|  |  |  | Age | 1.02 (1.01 to 1.03) | 0.002 | ** | 547 | 0.595 |
|  |  |  | L3 IMATI | 1.08 (0.99 to 1.17) | 0.080 |  | 547 | 0.595 |
|  |  |  | L3 SATI | 0.99 (0.98 to 1.01) | 0.296 |  | 547 | 0.595 |
|  |  |  | L3 SMI | 1.00 (0.98 to 1.02) | 0.938 |  | 547 | 0.595 |
|  |  |  | L3 VATI | 0.99 (0.97 to 1.00) | 0.128 |  | 547 | 0.595 |
|  |  |  | M1 | 2.47 (1.79 to 3.42) | <0.001 | *** | 547 | 0.595 |
|  |  |  | Sex (1=Male) | 1.19 (0.86 to 1.63) | 0.291 |  | 547 | 0.595 |
|  |  | Myosteatotic Fat Index (MFI) | Age | 1.02 (1.01 to 1.03) | 0.007 | ** | 547 | 0.616 |
|  |  |  | L3 IMATI | 0.97 (0.88 to 1.07) | 0.578 |  | 547 | 0.616 |
|  |  |  | L3 SATI | 1.00 (0.99 to 1.01) | 0.952 |  | 547 | 0.616 |
|  |  |  | L3 SMI | 1.00 (0.98 to 1.02) | 0.985 |  | 547 | 0.616 |
|  |  |  | L3 VATI | 1.00 (0.98 to 1.01) | 0.606 |  | 547 | 0.616 |
|  |  |  | M1 | 2.41 (1.76 to 3.30) | <0.001 | *** | 547 | 0.616 |
|  |  |  | Myosteatotic Fat Index (MFI) | 1.44 (1.22 to 1.70) | <0.001 | *** | 547 | 0.616 |
|  |  |  | Sex (1=Male) | 1.14 (0.86 to 1.52) | 0.372 |  | 547 | 0.616 |
|  |  | Sarcopenia Index (SI) | Age | 1.01 (0.99 to 1.02) | 0.286 |  | 547 | 0.624 |
|  |  |  | L3 IMATI | 0.98 (0.89 to 1.07) | 0.619 |  | 547 | 0.624 |
|  |  |  | L3 SATI | 1.00 (0.98 to 1.01) | 0.641 |  | 547 | 0.624 |
|  |  |  | L3 SMI | 1.00 (0.98 to 1.02) | 0.962 |  | 547 | 0.624 |
|  |  |  | L3 VATI | 1.00 (0.99 to 1.01) | 0.910 |  | 547 | 0.624 |
|  |  |  | M1 | 2.41 (1.76 to 3.29) | <0.001 | *** | 547 | 0.624 |
|  |  |  | Sarcopenia Index (SI) | 0.43 (0.30 to 0.60) | <0.001 | *** | 547 | 0.624 |
|  |  |  | Sex (1=Male) | 1.39 (1.04 to 1.86) | 0.028 | * | 547 | 0.624 |
| Clinical + ECOG + Single BCA | Age, Sex, M-Status, ECOG, Single BCA marker | Abdominal Fat Index (AFI) | Abdominal Fat Index (AFI) | 1.02 (0.69 to 1.51) | 0.933 |  | 542 | 0.62 |
|  |  |  | Age | 1.02 (1.01 to 1.03) | 0.002 | ** | 542 | 0.62 |
|  |  |  | ECOG | 1.40 (1.25 to 1.56) | <0.001 | *** | 542 | 0.62 |
|  |  |  | M1 | 2.26 (1.63 to 3.12) | <0.001 | *** | 542 | 0.62 |
|  |  |  | Sex (1=Male) | 1.25 (0.92 to 1.71) | 0.161 |  | 542 | 0.62 |
|  |  | Myosteatotic Fat Index (MFI) | Age | 1.02 (1.00 to 1.03) | 0.013 | * | 542 | 0.635 |
|  |  |  | ECOG | 1.31 (1.17 to 1.47) | <0.001 | *** | 542 | 0.635 |
|  |  |  | M1 | 2.28 (1.65 to 3.14) | <0.001 | *** | 542 | 0.635 |
|  |  |  | Myosteatotic Fat Index (MFI) | 1.33 (1.16 to 1.54) | <0.001 | *** | 542 | 0.635 |
|  |  |  | Sex (1=Male) | 1.18 (0.90 to 1.55) | 0.233 |  | 542 | 0.635 |
|  |  | Sarcopenia Index (SI) | Age | 1.01 (1.00 to 1.02) | 0.225 |  | 542 | 0.638 |
|  |  |  | ECOG | 1.30 (1.16 to 1.46) | <0.001 | *** | 542 | 0.638 |
|  |  |  | M1 | 2.31 (1.68 to 3.19) | <0.001 | *** | 542 | 0.638 |
|  |  |  | Sarcopenia Index (SI) | 0.49 (0.36 to 0.67) | <0.001 | *** | 542 | 0.638 |
|  |  |  | Sex (1=Male) | 1.42 (1.08 to 1.88) | 0.013 | * | 542 | 0.638 |
| Clinical + ECOG + BMI + Single BCA | Age, Sex, M-Status, ECOG, BMI, Single BCA marker | Abdominal Fat Index (AFI) | Abdominal Fat Index (AFI) | 1.01 (0.67 to 1.51) | 0.971 |  | 532 | 0.62 |
|  |  |  | Age | 1.02 (1.01 to 1.03) | 0.002 | ** | 532 | 0.62 |
|  |  |  | BMI | 0.99 (0.97 to 1.01) | 0.145 |  | 532 | 0.62 |
|  |  |  | ECOG | 1.38 (1.24 to 1.55) | <0.001 | *** | 532 | 0.62 |
|  |  |  | M1 | 2.31 (1.66 to 3.22) | <0.001 | *** | 532 | 0.62 |
|  |  |  | Sex (1=Male) | 1.25 (0.91 to 1.73) | 0.164 |  | 532 | 0.62 |
|  |  | Myosteatotic Fat Index (MFI) | Age | 1.02 (1.00 to 1.03) | 0.012 | * | 532 | 0.631 |
|  |  |  | BMI | 1.00 (0.98 to 1.01) | 0.647 |  | 532 | 0.631 |
|  |  |  | ECOG | 1.31 (1.16 to 1.47) | <0.001 | *** | 532 | 0.631 |
|  |  |  | M1 | 2.29 (1.65 to 3.18) | <0.001 | *** | 532 | 0.631 |
|  |  |  | Myosteatotic Fat Index (MFI) | 1.31 (1.13 to 1.53) | <0.001 | *** | 532 | 0.631 |
|  |  |  | Sex (1=Male) | 1.16 (0.88 to 1.54) | 0.288 |  | 532 | 0.631 |
|  |  | Sarcopenia Index (SI) | Age | 1.01 (1.00 to 1.02) | 0.224 |  | 532 | 0.635 |
|  |  |  | BMI | 1.00 (0.98 to 1.02) | 0.931 |  | 532 | 0.635 |
|  |  |  | ECOG | 1.30 (1.16 to 1.46) | <0.001 | *** | 532 | 0.635 |
|  |  |  | M1 | 2.28 (1.64 to 3.17) | <0.001 | *** | 532 | 0.635 |
|  |  |  | Sarcopenia Index (SI) | 0.50 (0.36 to 0.69) | <0.001 | *** | 532 | 0.635 |
|  |  |  | Sex (1=Male) | 1.39 (1.05 to 1.84) | 0.022 | * | 532 | 0.635 |
| Clinical + ECOG + L3 + Single BCA | Age, Sex, M-Status, ECOG, L3 indices, Single BCA marker | Abdominal Fat Index (AFI) | Abdominal Fat Index (AFI) | 1.00 (0.59 to 1.69) | 0.991 |  | 538 | 0.627 |
|  |  |  | Age | 1.02 (1.01 to 1.03) | 0.006 | ** | 538 | 0.627 |
|  |  |  | ECOG | 1.39 (1.24 to 1.55) | <0.001 | *** | 538 | 0.627 |
|  |  |  | L3 IMATI | 1.07 (0.98 to 1.17) | 0.121 |  | 538 | 0.627 |
|  |  |  | L3 SATI | 0.99 (0.97 to 1.00) | 0.158 |  | 538 | 0.627 |
|  |  |  | L3 SMI | 1.00 (0.99 to 1.02) | 0.689 |  | 538 | 0.627 |
|  |  |  | L3 VATI | 0.99 (0.98 to 1.01) | 0.219 |  | 538 | 0.627 |
|  |  |  | M1 | 2.26 (1.62 to 3.16) | <0.001 | *** | 538 | 0.627 |
|  |  |  | Sex (1=Male) | 1.26 (0.92 to 1.74) | 0.156 |  | 538 | 0.627 |
|  |  | Myosteatotic Fat Index (MFI) | Age | 1.02 (1.00 to 1.03) | 0.010 | ** | 538 | 0.634 |
|  |  |  | ECOG | 1.32 (1.17 to 1.48) | <0.001 | *** | 538 | 0.634 |
|  |  |  | L3 IMATI | 1.00 (0.90 to 1.11) | 0.982 |  | 538 | 0.634 |
|  |  |  | L3 SATI | 1.00 (0.98 to 1.01) | 0.526 |  | 538 | 0.634 |
|  |  |  | L3 SMI | 1.01 (0.99 to 1.02) | 0.632 |  | 538 | 0.634 |
|  |  |  | L3 VATI | 1.00 (0.98 to 1.01) | 0.455 |  | 538 | 0.634 |
|  |  |  | M1 | 2.27 (1.64 to 3.14) | <0.001 | *** | 538 | 0.634 |
|  |  |  | Myosteatotic Fat Index (MFI) | 1.27 (1.07 to 1.51) | 0.007 | ** | 538 | 0.634 |
|  |  |  | Sex (1=Male) | 1.19 (0.89 to 1.60) | 0.245 |  | 538 | 0.634 |
|  |  | Sarcopenia Index (SI) | Age | 1.01 (1.00 to 1.02) | 0.186 |  | 538 | 0.64 |
|  |  |  | ECOG | 1.31 (1.17 to 1.47) | <0.001 | *** | 538 | 0.64 |
|  |  |  | L3 IMATI | 0.99 (0.90 to 1.10) | 0.885 |  | 538 | 0.64 |
|  |  |  | L3 SATI | 0.99 (0.98 to 1.01) | 0.338 |  | 538 | 0.64 |
|  |  |  | L3 SMI | 1.01 (0.99 to 1.03) | 0.630 |  | 538 | 0.64 |
|  |  |  | L3 VATI | 1.00 (0.99 to 1.01) | 0.779 |  | 538 | 0.64 |
|  |  |  | M1 | 2.28 (1.65 to 3.16) | <0.001 | *** | 538 | 0.64 |
|  |  |  | Sarcopenia Index (SI) | 0.52 (0.37 to 0.75) | <0.001 | *** | 538 | 0.64 |
|  |  |  | Sex (1=Male) | 1.38 (1.03 to 1.86) | 0.033 | * | 538 | 0.64 |
| Clinical + ECOG + BMI + L3 + Single BCA | Age, Sex, M-Status, ECOG, BMI, L3 indices, Single BCA marker | Abdominal Fat Index (AFI) | Abdominal Fat Index (AFI) | 1.06 (0.63 to 1.80) | 0.825 |  | 532 | 0.626 |
|  |  |  | Age | 1.02 (1.01 to 1.03) | 0.006 | ** | 532 | 0.626 |
|  |  |  | BMI | 1.01 (0.98 to 1.03) | 0.613 |  | 532 | 0.626 |
|  |  |  | ECOG | 1.39 (1.24 to 1.55) | <0.001 | *** | 532 | 0.626 |
|  |  |  | L3 IMATI | 1.08 (0.99 to 1.18) | 0.104 |  | 532 | 0.626 |
|  |  |  | L3 SATI | 0.99 (0.97 to 1.01) | 0.158 |  | 532 | 0.626 |
|  |  |  | L3 SMI | 1.01 (0.99 to 1.03) | 0.570 |  | 532 | 0.626 |
|  |  |  | L3 VATI | 0.99 (0.97 to 1.01) | 0.166 |  | 532 | 0.626 |
|  |  |  | M1 | 2.28 (1.63 to 3.19) | <0.001 | *** | 532 | 0.626 |
|  |  |  | Sex (1=Male) | 1.21 (0.87 to 1.68) | 0.262 |  | 532 | 0.626 |
|  |  | Myosteatotic Fat Index (MFI) | Age | 1.02 (1.00 to 1.03) | 0.010 | ** | 532 | 0.633 |
|  |  |  | BMI | 1.01 (0.99 to 1.03) | 0.428 |  | 532 | 0.633 |
|  |  |  | ECOG | 1.32 (1.18 to 1.49) | <0.001 | *** | 532 | 0.633 |
|  |  |  | L3 IMATI | 1.01 (0.91 to 1.12) | 0.882 |  | 532 | 0.633 |
|  |  |  | L3 SATI | 0.99 (0.98 to 1.01) | 0.365 |  | 532 | 0.633 |
|  |  |  | L3 SMI | 1.01 (0.99 to 1.03) | 0.472 |  | 532 | 0.633 |
|  |  |  | L3 VATI | 0.99 (0.98 to 1.01) | 0.362 |  | 532 | 0.633 |
|  |  |  | M1 | 2.24 (1.61 to 3.12) | <0.001 | *** | 532 | 0.633 |
|  |  |  | Myosteatotic Fat Index (MFI) | 1.27 (1.06 to 1.51) | 0.009 | ** | 532 | 0.633 |
|  |  |  | Sex (1=Male) | 1.15 (0.85 to 1.56) | 0.372 |  | 532 | 0.633 |
|  |  | Sarcopenia Index (SI) | Age | 1.01 (1.00 to 1.02) | 0.210 |  | 532 | 0.641 |
|  |  |  | BMI | 1.02 (1.00 to 1.03) | 0.089 |  | 532 | 0.641 |
|  |  |  | ECOG | 1.31 (1.17 to 1.47) | <0.001 | *** | 532 | 0.641 |
|  |  |  | L3 IMATI | 1.00 (0.90 to 1.11) | 0.999 |  | 532 | 0.641 |
|  |  |  | L3 SATI | 0.99 (0.98 to 1.00) | 0.143 |  | 532 | 0.641 |
|  |  |  | L3 SMI | 1.01 (0.99 to 1.03) | 0.323 |  | 532 | 0.641 |
|  |  |  | L3 VATI | 1.00 (0.98 to 1.01) | 0.538 |  | 532 | 0.641 |
|  |  |  | M1 | 2.23 (1.60 to 3.09) | <0.001 | *** | 532 | 0.641 |
|  |  |  | Sarcopenia Index (SI) | 0.50 (0.35 to 0.72) | <0.001 | *** | 532 | 0.641 |
|  |  |  | Sex (1=Male) | 1.31 (0.97 to 1.78) | 0.077 |  | 532 | 0.641 |

Sig.: * p<0.05, ** p<0.01, *** p<0.001.

*Supplementary Table S1. Complete multivariable Cox proportional hazards regression results. Hazard ratios (HR), 95% confidence intervals and p-values for every covariate in each model specification referenced in the main text: the per-marker univariate models, the clinical reference models (age, sex, M-status; and the same plus ECOG), the per-marker staged adjustments (+BMI; +L3 indices; +ECOG; and their combinations up to the fully adjusted model), and the combined models entering all three volumetric indices simultaneously at each adjustment level. Model rows are grouped: the Model, Covariates and Model Context labels apply to every row beneath them until the next label. N is the complete-case sample size of that fit and C-index is Harrell's concordance for the model as a whole, not for the individual covariate. Estimates are shown to two decimals; values are rounded from the three-decimal estimates in the underlying analysis.*

# Supplementary Table S2. Era-stratified overall survival

| Stratum | Era | n | Events | Median OS, months (95% CI) | Max follow-up, months | 1-year survival, % (95% CI) | 3-year survival, % (95% CI) | 5-year survival, % (95% CI) |
| --- | --- | --- | --- | --- | --- | --- | --- | --- |
| Overall | Pre-RAMIE | 291 | 228 | 28.8 (22.2 to 35.3) | 172.0 | 68.4 (62.7 to 73.4) | 44.3 (38.6 to 49.9) | 34.0 (28.6 to 39.5) |
| Overall | RAMIE | 267 | 139 | 26.3 (21.4 to 39.6) | 73.3 | 74.4 (68.2 to 78.8) | 45.7 (38.8 to 52.3) | 34.2 (26.4 to 42.2) |
| Sex=F | Pre-RAMIE | 60 | 49 | 31.6 (9.5 to 54.0) | 172.0 | 61.7 (46.5 to 71.1) | 45.0 (32.2 to 57.0) | 35.0 (23.3 to 47.0) |
| Sex=F | RAMIE | 52 | 21 | 49.2 (29.2 to NR) | 72.6 | 76.8 (60.6 to 84.5) | 58.5 (42.2 to 71.7) | 47.9 (29.2 to 64.3) |
| Sex=M | Pre-RAMIE | 231 | 179 | 28.4 (21.6 to 36.8) | 171.0 | 70.1 (63.8 to 75.6) | 44.2 (37.3 to 50.0) | 33.8 (27.7 to 39.9) |
| Sex=M | RAMIE | 215 | 118 | 23.3 (20.1 to 36.6) | 73.3 | 73.8 (66.8 to 78.7) | 42.7 (35.2 to 50.1) | 30.7 (22.1 to 39.6) |
| Sex=F/M0 | Pre-RAMIE | 52 | 41 | 35.3 (12.8 to 64.0) | 172.0 | 65.4 (48.9 to 74.9) | 50.0 (35.8 to 62.6) | 38.5 (25.4 to 51.3) |
| Sex=F/M0 | RAMIE | 48 | 18 | 49.2 (30.7 to NR) | 72.6 | 81.1 (64.4 to 88.1) | 61.1 (43.7 to 74.6) | 50.0 (30.2 to 66.9) |
| Sex=F/M1 | Pre-RAMIE | 8 | 8 | 7.6 (2.9 to 33.2) | 74.8 | 37.5 (8.7 to 67.4) | 12.5 (0.7 to 42.3) | 12.5 (NE) |
| Sex=M/M0 | Pre-RAMIE | 202 | 153 | 30.9 (24.2 to 45.4) | 171.0 | 73.3 (66.6 to 78.8) | 48.5 (41.0 to 54.7) | 37.1 (30.5 to 43.8) |
| Sex=M/M0 | RAMIE | 199 | 106 | 25.4 (20.3 to 38.8) | 73.3 | 75.2 (68.6 to 80.7) | 44.0 (36.1 to 51.7) | 31.6 (22.5 to 41.2) |
| Sex=M/M1 | Pre-RAMIE | 29 | 26 | 9.1 (4.7 to 15.4) | 157.5 | 48.3 (26.5 to 61.6) | 13.8 (4.3 to 28.6) | 10.3 (2.6 to 24.3) |
| Sex=M/M1 | RAMIE | 16 | 12 | 12.0 (3.5 to 36.6) | 64.6 | 55.6 (22.9 to 70.3) | 26.0 (3.1 to 41.4) | 17.4 (3.1 to 41.4) |

*Supplementary Table S2. Era-stratified overall survival. Patients are split at the institutional transition to robot-assisted minimally invasive esophagectomy (RAMIE) on 1 July 2019. The cohort comprises the 558 patients with a recorded diagnosis date and non-negative follow-up time (291 Pre-RAMIE, 267 RAMIE). For each stratum the table reports n, events, median overall survival and 1-, 3- and 5-year survival probabilities, each with a 95% confidence interval. NR denotes a limit not reached within follow-up and NE an interval that is not estimable. The female metastatic RAMIE cell (n=4) is omitted because no events allow a survival estimate; its size is given in Supplementary Table S3.*

# Supplementary Table S3. Log-rank tests, Pre-RAMIE vs. RAMIE

**Panel A. Full cohort**

| Stratum | n Pre-RAMIE | n RAMIE | Log-rank chi-square | p |
| --- | --- | --- | --- | --- |
| Overall | 291 | 267 | 0.168 | 0.682 |
| Sex=F | 60 | 52 | 2.884 | 0.089 |
| Sex=M | 231 | 215 | 0.185 | 0.667 |
| Sex=F/M0 | 52 | 48 | 2.498 | 0.114 |
| Sex=F/M1 | 8 | 4 | not computed (n<5 per era) | not computed (n<5 per era) |
| Sex=M/M0 | 202 | 199 | 1.046 | 0.306 |
| Sex=M/M1 | 29 | 16 | 0.371 | 0.543 |

**Panel B. Sensitivity window, 1 July 2019 +/- 2 years**

| Stratum | n Pre-RAMIE | n RAMIE | Log-rank chi-square | p |
| --- | --- | --- | --- | --- |
| Overall | 75 | 81 | 0.015 | 0.903 |
| Sex=F | 18 | 19 | 0.030 | 0.862 |
| Sex=M | 57 | 62 | 0.066 | 0.797 |
| Sex=F/M0 | 16 | 17 | 0.367 | 0.545 |
| Sex=F/M1 | 2 | 2 | not computed (n<5 per era) | not computed (n<5 per era) |
| Sex=M/M0 | 49 | 55 | 0.790 | 0.374 |
| Sex=M/M1 | 8 | 7 | 4.170 | 0.041 |

*Supplementary Table S3. Log-rank tests comparing Pre-RAMIE and RAMIE overall survival. Panel A covers the full era cohort (n=558), stratified by sex and by sex x M-status. Panel B restricts the comparison to diagnoses within two years either side of 1 July 2019, as a sensitivity analysis against case-mix drift over longer horizons. Strata with fewer than five patients in an era are listed with their sample sizes but no test statistic. The Sex=M/M1 cell in Panel B reaches p=0.041 on 8 versus 7 patients; it is not corrected for multiple testing and is not interpreted as an era effect.*

# Supplementary Table S4. Case-mix across eras

**Panel A. Distributions**

| Variable | Level | n Pre-RAMIE | n RAMIE | Pre-RAMIE | RAMIE |
| --- | --- | --- | --- | --- | --- |
| Age (years) | median [IQR] (mean +/- SD) | 294 | 270 | 64.0 [57.2-70.0] (63.3 +/- 10.3) | 65.0 [59.0-72.0] (65.0 +/- 9.7) |
| Sex | F | 60 | 52 | 60 (20.4%) | 52 (19.3%) |
| Sex | M | 234 | 218 | 234 (79.6%) | 218 (80.7%) |
| M1 status | False | 257 | 250 | 257 (87.4%) | 250 (92.6%) |
| M1 status | True | 37 | 20 | 37 (12.6%) | 20 (7.4%) |
| ECOG | 0.0 | 105 | 91 | 105 (36.8%) | 91 (34.6%) |
| ECOG | 1.0 | 112 | 139 | 112 (39.3%) | 139 (52.9%) |
| ECOG | 2.0 | 40 | 26 | 40 (14.0%) | 26 (9.9%) |
| ECOG | 3.0 | 24 | 6 | 24 (8.4%) | 6 (2.3%) |
| ECOG | 4.0 | 4 | 1 | 4 (1.4%) | 1 (0.4%) |

**Panel B. Two-sample tests**

| Variable | Test | Statistic | p | n Pre-RAMIE | n RAMIE |
| --- | --- | --- | --- | --- | --- |
| Age (years) | Mann-Whitney U | 36033.000 | 0.058 | 294 | 270 |
| Sex | chi-square | 0.056 | 0.813 | 294 | 270 |
| M1 status | chi-square | 3.603 | 0.058 | 294 | 270 |
| ECOG | chi-square | 18.621 | 0.0009 | 285 | 263 |

*Supplementary Table S4. Case-mix comparison across the Pre-RAMIE and RAMIE eras. Panel A reports the distribution of age, sex, M-status and ECOG performance status; Panel B the corresponding two-sample tests (Mann-Whitney U for age, chi-square otherwise). This table is computed on all 564 patients with a recorded diagnosis date, and so uses a larger denominator than Supplementary Tables S2 and S3, which additionally require non-negative follow-up time (n=558). Per-variable denominators differ further where a value is missing; ECOG is available for 548. ECOG distribution differs between eras (p=0.0009, fewer ECOG 2-4 in the RAMIE era), M1 prevalence (12.6% vs. 7.4%, p=0.058) and median age (p=0.058) are borderline, and sex is unchanged. The era sensitivity Cox specifications in Supplementary Table S5 adjust for these shifts.*

# Supplementary Table S5. Cox sensitivity to era specification

**S5a. Primary specification (base + ECOG, n=542)**

| Marker | Specification | Hazard ratio | p | Change vs reference, % |
| --- | --- | --- | --- | --- |
| Abdominal Fat Index | reference | 1.02 | 0.933 |  |
| Abdominal Fat Index | +era | 1.02 | 0.919 | +0.4 |
| Abdominal Fat Index | strat-era | 1.02 | 0.904 | +0.7 |
| Myosteatotic Fat Index | reference | 1.33 | <0.001 |  |
| Myosteatotic Fat Index | +era | 1.34 | <0.001 | +0.5 |
| Myosteatotic Fat Index | strat-era | 1.34 | <0.001 | +0.7 |
| Sarcopenia Index | reference | 0.49 | <0.001 |  |
| Sarcopenia Index | +era | 0.49 | <0.001 | -0.3 |
| Sarcopenia Index | strat-era | 0.49 | <0.001 | +0.1 |

**S5b. Secondary specification (base, n=557)**

| Marker | Specification | Hazard ratio | p | Change vs reference, % |
| --- | --- | --- | --- | --- |
| Abdominal Fat Index | reference | 1.02 | 0.924 |  |
| Abdominal Fat Index | +era | 1.02 | 0.936 | -0.3 |
| Abdominal Fat Index | strat-era | 1.02 | 0.913 | +0.3 |
| Myosteatotic Fat Index | reference | 1.47 | <0.001 |  |
| Myosteatotic Fat Index | +era | 1.48 | <0.001 | +0.2 |
| Myosteatotic Fat Index | strat-era | 1.48 | <0.001 | +0.5 |
| Sarcopenia Index | reference | 0.40 | <0.001 |  |
| Sarcopenia Index | +era | 0.40 | <0.001 | -0.1 |
| Sarcopenia Index | strat-era | 0.40 | <0.001 | +0.1 |

*Supplementary Table S5. Cox sensitivity analyses of era specifications. For each marker the hazard ratio is reported under three specifications: reference, being age, sex, M-status, ECOG plus marker in S5a and age, sex, M-status plus marker in S5b; +era, adding a binary Pre-RAMIE/RAMIE indicator at the 1 July 2019 cutpoint; and strat-era, stratifying the baseline hazard on era. The last column gives the percentage change in the marker hazard ratio relative to the reference row for the same marker. S5a (n=542) is the primary specification used in the main text and S5b (n=557) is shown for completeness. Across both sub-tables every marker hazard ratio moves by less than 1% with no change in significance. The continuous calendar-time specification is reported separately in Supplementary Table S6.*

# Supplementary Table S6. Calendar-time trend

| Cohort | HR per +1 calendar year (95% CI) | p | Harrell C-index | n |
| --- | --- | --- | --- | --- |
| Base (age, sex, M-status) | 1.001 (0.974 to 1.029) | 0.921 | 0.582 | 557 |
| Base + ECOG | 1.010 (0.982 to 1.040) | 0.482 | 0.620 | 542 |

*Supplementary Table S6. Calendar-time trend in overall survival. Hazard ratio per additional calendar year from the adjusted Cox model with diagnosis date entered as a continuous covariate (years since 1 January 2011, no cutpoint), for the base specification (age, sex, M-status; n=557) and the primary adjusted specification (age, sex, M-status, ECOG; n=542). Harrell's C-index is for the model as a whole. Calendar time is not independently prognostic in either specification, consistent with the absence of an era effect in Supplementary Tables S2 to S5.*

# Supplementary Table S7. Incremental discrimination (C-index)

| Adjustment cohort | Marker | Reference model | n (events) | C-index, nested (95% CI) | C-index, full (95% CI) | Delta C-index (95% CI) | LR chi-square (df=1) | LR p |
| --- | --- | --- | --- | --- | --- | --- | --- | --- |
| Base (age, sex, M-status) | Sarcopenia Index | vs. clinical | 557 (367) | 0.582 (0.576 to 0.583) | 0.625 (0.616 to 0.626) | +0.0436 (+0.0353 to +0.0469) | 37.166 | <0.001 |
| Base (age, sex, M-status) | Sarcopenia Index | vs. clinical + BMI | 541 (353) | 0.592 (0.568 to 0.597) | 0.621 (0.610 to 0.621) | +0.0290 (+0.0185 to +0.0482) | 29.492 | <0.001 |
| Base (age, sex, M-status) | Sarcopenia Index | vs. clinical + L3 | 547 (358) | 0.594 (0.582 to 0.602) | 0.624 (0.610 to 0.625) | +0.0298 (+0.0185 to +0.0334) | 24.555 | <0.001 |
| Base (age, sex, M-status) | Sarcopenia Index | vs. clinical + BMI + L3 | 541 (353) | 0.592 (0.579 to 0.602) | 0.623 (0.607 to 0.625) | +0.0308 (+0.0154 to +0.0347) | 24.551 | <0.001 |
| Base (age, sex, M-status) | Myosteatotic Fat Index | vs. clinical | 557 (367) | 0.582 (0.576 to 0.583) | 0.621 (0.611 to 0.622) | +0.0387 (+0.0313 to +0.0418) | 27.327 | <0.001 |
| Base (age, sex, M-status) | Myosteatotic Fat Index | vs. clinical + BMI | 541 (353) | 0.592 (0.568 to 0.597) | 0.614 (0.604 to 0.616) | +0.0226 (+0.0125 to +0.0410) | 22.321 | <0.001 |
| Base (age, sex, M-status) | Myosteatotic Fat Index | vs. clinical + L3 | 547 (358) | 0.594 (0.582 to 0.601) | 0.616 (0.601 to 0.618) | +0.0217 (+0.0117 to +0.0252) | 16.383 | <0.001 |
| Base (age, sex, M-status) | Myosteatotic Fat Index | vs. clinical + BMI + L3 | 541 (353) | 0.592 (0.580 to 0.602) | 0.615 (0.598 to 0.616) | +0.0221 (+0.0078 to +0.0231) | 15.222 | <0.001 |
| Base (age, sex, M-status) | Abdominal Fat Index | vs. clinical | 557 (367) | 0.582 (0.576 to 0.583) | 0.582 (0.569 to 0.586) | +0.0002 (-0.0098 to +0.0032) | 0.009 | 0.924 |
| Base (age, sex, M-status) | Abdominal Fat Index | vs. clinical + BMI | 541 (353) | 0.592 (0.567 to 0.596) | 0.592 (0.566 to 0.598) | <0.0001 (-0.0130 to +0.0031) | 0.001 | 0.970 |
| Base (age, sex, M-status) | Abdominal Fat Index | vs. clinical + L3 | 547 (358) | 0.594 (0.582 to 0.602) | 0.595 (0.579 to 0.600) | +0.0014 (-0.0074 to +0.0015) | 0.099 | 0.753 |
| Base (age, sex, M-status) | Abdominal Fat Index | vs. clinical + BMI + L3 | 541 (353) | 0.592 (0.579 to 0.602) | 0.593 (0.577 to 0.600) | +0.0006 (-0.0080 to +0.0017) | 0.211 | 0.646 |
| Base + ECOG | Sarcopenia Index | vs. clinical | 542 (353) | 0.620 (0.613 to 0.621) | 0.638 (0.628 to 0.639) | +0.0178 (+0.0127 to +0.0198) | 21.318 | <0.001 |
| Base + ECOG | Sarcopenia Index | vs. clinical + BMI | 532 (344) | 0.620 (0.609 to 0.623) | 0.635 (0.623 to 0.637) | +0.0157 (+0.0078 to +0.0216) | 17.328 | <0.001 |
| Base + ECOG | Sarcopenia Index | vs. clinical + L3 | 538 (349) | 0.627 (0.615 to 0.628) | 0.640 (0.626 to 0.640) | +0.0134 (+0.0072 to +0.0158) | 13.119 | <0.001 |
| Base + ECOG | Sarcopenia Index | vs. clinical + BMI + L3 | 532 (344) | 0.625 (0.611 to 0.626) | 0.641 (0.623 to 0.639) | +0.0153 (+0.0070 to +0.0176) | 13.916 | <0.001 |
| Base + ECOG | Myosteatotic Fat Index | vs. clinical | 542 (353) | 0.620 (0.612 to 0.621) | 0.635 (0.625 to 0.636) | +0.0151 (+0.0095 to +0.0176) | 14.910 | <0.001 |
| Base + ECOG | Myosteatotic Fat Index | vs. clinical + BMI | 532 (344) | 0.620 (0.609 to 0.623) | 0.631 (0.618 to 0.633) | +0.0108 (+0.0043 to +0.0168) | 11.951 | <0.001 |
| Base + ECOG | Myosteatotic Fat Index | vs. clinical + L3 | 538 (349) | 0.627 (0.615 to 0.628) | 0.634 (0.620 to 0.635) | +0.0073 (+0.0009 to +0.0104) | 6.806 | 0.009 |
| Base + ECOG | Myosteatotic Fat Index | vs. clinical + BMI + L3 | 532 (344) | 0.625 (0.610 to 0.626) | 0.633 (0.615 to 0.634) | +0.0074 (+0.0000 to +0.0107) | 6.515 | 0.011 |
| Base + ECOG | Abdominal Fat Index | vs. clinical | 542 (353) | 0.620 (0.612 to 0.621) | 0.620 (0.607 to 0.621) | -0.0001 (-0.0100 to +0.0010) | 0.007 | 0.933 |
| Base + ECOG | Abdominal Fat Index | vs. clinical + BMI | 532 (344) | 0.620 (0.609 to 0.623) | 0.620 (0.605 to 0.623) | -0.0002 (-0.0109 to +0.0013) | 0.001 | 0.971 |
| Base + ECOG | Abdominal Fat Index | vs. clinical + L3 | 538 (349) | 0.627 (0.614 to 0.628) | 0.627 (0.611 to 0.628) | <0.0001 (-0.0087 to +0.0009) | 0.0001 | 0.991 |
| Base + ECOG | Abdominal Fat Index | vs. clinical + BMI + L3 | 532 (344) | 0.625 (0.611 to 0.626) | 0.626 (0.608 to 0.626) | +0.0002 (-0.0088 to +0.0014) | 0.048 | 0.826 |

*Supplementary Table S7. Incremental discrimination of each volumetric body composition index added to nested reference Cox models. Rows enumerate every combination of adjustment cohort, marker and reference model that was evaluated. The reference models are: vs. clinical (age, sex, M-status, and ECOG in the base + ECOG cohort); vs. clinical + BMI; vs. clinical + L3 (the four L3 indices); and vs. clinical + BMI + L3. For each the table reports Harrell's C-index of the nested model and of the full model (nested plus the volumetric marker), the change in C-index, and a likelihood-ratio test. Ninety-five percent confidence intervals are paired percentile bootstrap (B=500): both models are refit on each resample and Harrell's C is evaluated on the original sample, avoiding the tie deflation that in-sample evaluation on duplicated bootstrap rows would produce; resamples with fewer than five events or non-convergent fits are dropped. The contrast cited in the main text is base + ECOG with vs. clinical + BMI + L3 (nested model: age, sex, M-status, ECOG, BMI, four L3 indices; n=532). Time-dependent AUC was not computed.*

# Supplementary Table S8. Sex x marker interactions and subgroup counts

**S8a. Subgroup composition**

| Subgroup | Sex | M-status | n | Events | Median follow-up, months |
| --- | --- | --- | --- | --- | --- |
| Female, M0 | Female | M0 | 96 | 56 | 29.5 |
| Female, M1 | Female | M1 | 11 | 10 | 5.2 |
| Female, all | Female | All | 107 | 66 | 25.4 |
| Male, M0 | Male | M0 | 394 | 253 | 22.4 |
| Male, M1 | Male | M1 | 41 | 34 | 12.0 |
| Male, all | Male | All | 435 | 287 | 21.7 |

**S8b. Sex x marker interaction terms**

| Marker | Cohort | n (events) | Marker HR (95% CI) | Marker p | Interaction HR, male vs. female (95% CI) | Interaction Wald p | LR chi-square (df=1) | LR p |
| --- | --- | --- | --- | --- | --- | --- | --- | --- |
| Sarcopenia Index | Base (age, sex, M-status) | 557 (367) | 0.40 (0.30 to 0.54) | <0.001 | 2.36 (1.17 to 4.75) | 0.016 | 6.006 | 0.014 |
| Myosteatotic Fat Index | Base (age, sex, M-status) | 557 (367) | 1.47 (1.28 to 1.69) | <0.001 | 0.75 (0.54 to 1.03) | 0.079 | 2.994 | 0.084 |
| Abdominal Fat Index | Base (age, sex, M-status) | 557 (367) | 1.02 (0.69 to 1.51) | 0.924 | 0.26 (0.06 to 1.10) | 0.066 | 3.129 | 0.077 |
| Sarcopenia Index | Base + ECOG | 542 (353) | 0.49 (0.36 to 0.67) | <0.001 | 2.23 (1.09 to 4.56) | 0.029 | 4.970 | 0.026 |
| Myosteatotic Fat Index | Base + ECOG | 542 (353) | 1.33 (1.16 to 1.54) | <0.001 | 0.72 (0.52 to 1.00) | 0.048 | 3.747 | 0.053 |
| Abdominal Fat Index | Base + ECOG | 542 (353) | 1.02 (0.69 to 1.51) | 0.933 | 0.34 (0.08 to 1.51) | 0.155 | 1.934 | 0.164 |

**S8c. Sex-stratified hazard ratios**

| Marker | Cohort | Female n (events) | Female-only HR (95% CI) | Female-only p | Male n (events) | Male-only HR (95% CI) | Male-only p |
| --- | --- | --- | --- | --- | --- | --- | --- |
| Sarcopenia Index | Base (age, sex, M-status) | 111 (70) | 0.21 (0.11 to 0.40) | <0.001 | 446 (297) | 0.49 (0.35 to 0.68) | <0.001 |
| Myosteatotic Fat Index | Base (age, sex, M-status) | 111 (70) | 1.84 (1.38 to 2.46) | <0.001 | 446 (297) | 1.39 (1.19 to 1.63) | <0.001 |
| Abdominal Fat Index | Base (age, sex, M-status) | 111 (70) | 4.35 (0.97 to 19.45) | 0.054 | 446 (297) | 0.94 (0.62 to 1.41) | 0.750 |
| Sarcopenia Index | Base + ECOG | 107 (66) | 0.25 (0.12 to 0.49) | <0.001 | 435 (287) | 0.60 (0.42 to 0.84) | 0.003 |
| Myosteatotic Fat Index | Base + ECOG | 107 (66) | 1.76 (1.30 to 2.38) | <0.001 | 435 (287) | 1.24 (1.05 to 1.46) | 0.010 |
| Abdominal Fat Index | Base + ECOG | 107 (66) | 3.59 (0.77 to 16.72) | 0.104 | 435 (287) | 0.95 (0.63 to 1.43) | 0.805 |

*Supplementary Table S8. Sex x marker interaction analyses and subgroup composition. S8a gives sample sizes, events and median follow-up for the four sex x M-status subgroups of the complete-case adjusted cohort (n=542, 353 events), with the sex totals shown for reference. S8b reports the formal sex x marker interaction fitted on two cohorts: base (age, sex, M-status plus marker; n=557) and base + ECOG (the primary adjusted model used in the main text; n=542). For each it gives the marker main effect, the interaction hazard ratio for males relative to females, and the likelihood-ratio test against the corresponding main-effects model. S8c reports the hazard ratios obtained by refitting the same model separately within each sex, for both cohorts. Values quoted in the main text are the base + ECOG rows. On that cohort the Sarcopenia Index x sex interaction is significant (p=0.026), the Myosteatotic Fat Index x sex interaction is borderline (p=0.053) and the Abdominal Fat Index x sex interaction is not supported (p=0.164). Three-way sex x M-status x marker interactions were not fitted because the female metastatic cell (n=11, 10 events) is too small.*

# Supplementary Table S9. Proportional hazards diagnostics

| Model | Covariate | Chi-square | p | PH violated (p<0.05) |
| --- | --- | --- | --- | --- |
| Primary adjusted + Sarcopenia Index | Age | 3.963 | 0.046 | yes |
| Primary adjusted + Sarcopenia Index | BMI | 1.279 | 0.258 | no |
| Primary adjusted + Sarcopenia Index | ECOG | 0.135 | 0.714 | no |
| Primary adjusted + Sarcopenia Index | Sex (male) | 4.540 | 0.033 | yes |
| Primary adjusted + Sarcopenia Index | M1 status | 2.235 | 0.135 | no |
| Primary adjusted + Sarcopenia Index | L3 IMATI | 0.301 | 0.583 | no |
| Primary adjusted + Sarcopenia Index | L3 SATI | 2.711 | 0.100 | no |
| Primary adjusted + Sarcopenia Index | L3 SMI | 1.374 | 0.241 | no |
| Primary adjusted + Sarcopenia Index | L3 VATI | 3.480 | 0.062 | no |
| Primary adjusted + Sarcopenia Index | Sarcopenia Index | 0.627 | 0.428 | no |
| Primary adjusted + Myosteatotic Fat Index | Age | 3.417 | 0.065 | no |
| Primary adjusted + Myosteatotic Fat Index | BMI | 2.437 | 0.118 | no |
| Primary adjusted + Myosteatotic Fat Index | ECOG | 0.431 | 0.511 | no |
| Primary adjusted + Myosteatotic Fat Index | Sex (male) | 5.581 | 0.018 | yes |
| Primary adjusted + Myosteatotic Fat Index | M1 status | 1.753 | 0.186 | no |
| Primary adjusted + Myosteatotic Fat Index | L3 IMATI | 0.945 | 0.331 | no |
| Primary adjusted + Myosteatotic Fat Index | L3 SATI | 3.988 | 0.046 | yes |
| Primary adjusted + Myosteatotic Fat Index | L3 SMI | 1.068 | 0.301 | no |
| Primary adjusted + Myosteatotic Fat Index | L3 VATI | 2.585 | 0.108 | no |
| Primary adjusted + Myosteatotic Fat Index | Myosteatotic Fat Index | 0.025 | 0.874 | no |
| Primary adjusted + Abdominal Fat Index | Age | 4.011 | 0.045 | yes |
| Primary adjusted + Abdominal Fat Index | BMI | 5.586 | 0.018 | yes |
| Primary adjusted + Abdominal Fat Index | ECOG | 0.235 | 0.627 | no |
| Primary adjusted + Abdominal Fat Index | Sex (male) | 14.870 | <0.001 | yes |
| Primary adjusted + Abdominal Fat Index | M1 status | 4.101 | 0.043 | yes |
| Primary adjusted + Abdominal Fat Index | Abdominal Fat Index | 13.351 | <0.001 | yes |
| Primary adjusted + Abdominal Fat Index | L3 IMATI | 1.685 | 0.194 | no |
| Primary adjusted + Abdominal Fat Index | L3 SATI | 0.036 | 0.849 | no |
| Primary adjusted + Abdominal Fat Index | L3 SMI | 0.142 | 0.706 | no |
| Primary adjusted + Abdominal Fat Index | L3 VATI | 0.859 | 0.354 | no |
| Combined fully adjusted | Age | 3.983 | 0.046 | yes |
| Combined fully adjusted | BMI | 1.817 | 0.178 | no |
| Combined fully adjusted | ECOG | 0.137 | 0.712 | no |
| Combined fully adjusted | Sex (male) | 9.573 | 0.002 | yes |
| Combined fully adjusted | M1 status | 5.188 | 0.023 | yes |
| Combined fully adjusted | Abdominal Fat Index | 12.169 | <0.001 | yes |
| Combined fully adjusted | L3 IMATI | 0.444 | 0.505 | no |
| Combined fully adjusted | L3 SATI | 0.035 | 0.852 | no |
| Combined fully adjusted | L3 SMI | 0.796 | 0.372 | no |
| Combined fully adjusted | L3 VATI | 0.189 | 0.663 | no |
| Combined fully adjusted | Myosteatotic Fat Index | 0.600 | 0.439 | no |
| Combined fully adjusted | Sarcopenia Index | 0.791 | 0.374 | no |

*Supplementary Table S9. Proportional hazards diagnostics for the per-marker primary adjusted Cox models (age, sex, M-status, ECOG, BMI and the four L3 indices plus one volumetric marker) and for the combined fully adjusted model. The table reports the scaled Schoenfeld residual chi-square statistic and p-value for each covariate, with time transformed by ranks; each test has one degree of freedom. Covariates flagged at p<0.05 are age, sex, M-status, BMI in the Abdominal Fat Index model, L3 SATI in the Myosteatotic Fat Index model, and the Abdominal Fat Index itself. The Sarcopenia Index and the Myosteatotic Fat Index satisfy the proportional hazards assumption in every model. The Abdominal Fat Index is not significant in any adjusted analysis, so its time-varying behaviour does not affect the inferential claims.*

# Supplementary Table S10. Collinearity: correlation and VIF

**Panel A. Pearson correlation matrix**

| Predictor | Age | BMI | L3 SMI | L3 SATI | L3 VATI | L3 IMATI | Sarcopenia Index | Myosteatotic Fat Index | Abdominal Fat Index |
| --- | --- | --- | --- | --- | --- | --- | --- | --- | --- |
| Age | 1.00 | -0.03 | -0.12 | -0.06 | 0.06 | 0.14 | -0.42 | 0.21 | 0.12 |
| BMI | -0.03 | 1.00 | 0.13 | 0.36 | 0.34 | 0.25 | 0.28 | -0.20 | -0.03 |
| L3 SMI | -0.12 | 0.13 | 1.00 | 0.69 | 0.73 | 0.62 | 0.32 | -0.28 | 0.05 |
| L3 SATI | -0.06 | 0.36 | 0.69 | 1.00 | 0.75 | 0.76 | 0.25 | -0.36 | -0.23 |
| L3 VATI | 0.06 | 0.34 | 0.73 | 0.75 | 1.00 | 0.76 | 0.29 | -0.30 | 0.24 |
| L3 IMATI | 0.14 | 0.25 | 0.62 | 0.76 | 0.76 | 1.00 | -0.03 | 0.01 | 0.02 |
| Sarcopenia Index | -0.42 | 0.28 | 0.32 | 0.25 | 0.29 | -0.03 | 1.00 | -0.58 | 0.03 |
| Myosteatotic Fat Index | 0.21 | -0.20 | -0.28 | -0.36 | -0.30 | 0.01 | -0.58 | 1.00 | 0.10 |
| Abdominal Fat Index | 0.12 | -0.03 | 0.05 | -0.23 | 0.24 | 0.02 | 0.03 | 0.10 | 1.00 |

**Panel B. Variance inflation factors**

| Model | Predictor | VIF | Flag |
| --- | --- | --- | --- |
| Primary adjusted + Sarcopenia Index | Age | 1.33 | ok |
| Primary adjusted + Sarcopenia Index | Sex (male) | 1.25 | ok |
| Primary adjusted + Sarcopenia Index | M1 status | 1.03 | ok |
| Primary adjusted + Sarcopenia Index | ECOG | 1.12 | ok |
| Primary adjusted + Sarcopenia Index | BMI | 1.34 | ok |
| Primary adjusted + Sarcopenia Index | L3 SMI | 2.84 | ok |
| Primary adjusted + Sarcopenia Index | L3 SATI | 3.94 | ok |
| Primary adjusted + Sarcopenia Index | L3 VATI | 4.06 | ok |
| Primary adjusted + Sarcopenia Index | L3 IMATI | 3.83 | ok |
| Primary adjusted + Sarcopenia Index | Sarcopenia Index | 1.96 | ok |
| Primary adjusted + Myosteatotic Fat Index | Age | 1.18 | ok |
| Primary adjusted + Myosteatotic Fat Index | Sex (male) | 1.22 | ok |
| Primary adjusted + Myosteatotic Fat Index | M1 status | 1.03 | ok |
| Primary adjusted + Myosteatotic Fat Index | ECOG | 1.10 | ok |
| Primary adjusted + Myosteatotic Fat Index | BMI | 1.29 | ok |
| Primary adjusted + Myosteatotic Fat Index | L3 SMI | 2.81 | ok |
| Primary adjusted + Myosteatotic Fat Index | L3 SATI | 4.42 | ok |
| Primary adjusted + Myosteatotic Fat Index | L3 VATI | 4.03 | ok |
| Primary adjusted + Myosteatotic Fat Index | L3 IMATI | 4.17 | ok |
| Primary adjusted + Myosteatotic Fat Index | Myosteatotic Fat Index | 1.69 | ok |
| Primary adjusted + Abdominal Fat Index | Age | 1.18 | ok |
| Primary adjusted + Abdominal Fat Index | Sex (male) | 1.40 | ok |
| Primary adjusted + Abdominal Fat Index | M1 status | 1.04 | ok |
| Primary adjusted + Abdominal Fat Index | ECOG | 1.05 | ok |
| Primary adjusted + Abdominal Fat Index | BMI | 1.29 | ok |
| Primary adjusted + Abdominal Fat Index | L3 SMI | 2.83 | ok |
| Primary adjusted + Abdominal Fat Index | L3 SATI | 5.15 | moderate |
| Primary adjusted + Abdominal Fat Index | L3 VATI | 5.20 | moderate |
| Primary adjusted + Abdominal Fat Index | L3 IMATI | 3.14 | ok |
| Primary adjusted + Abdominal Fat Index | Abdominal Fat Index | 2.10 | ok |
| Combined fully adjusted | Age | 1.34 | ok |
| Combined fully adjusted | Sex (male) | 1.47 | ok |
| Combined fully adjusted | M1 status | 1.05 | ok |
| Combined fully adjusted | ECOG | 1.14 | ok |
| Combined fully adjusted | BMI | 1.35 | ok |
| Combined fully adjusted | L3 SMI | 2.85 | ok |
| Combined fully adjusted | L3 SATI | 5.68 | moderate |
| Combined fully adjusted | L3 VATI | 5.72 | moderate |
| Combined fully adjusted | L3 IMATI | 4.41 | ok |
| Combined fully adjusted | Sarcopenia Index | 2.29 | ok |
| Combined fully adjusted | Myosteatotic Fat Index | 1.97 | ok |
| Combined fully adjusted | Abdominal Fat Index | 2.11 | ok |

*Supplementary Table S10. Collinearity diagnostics for the continuous predictors used in the primary adjusted and fully adjusted Cox models. Panel A is the Pearson correlation matrix among age, BMI, the four L3 reference indices and the three volumetric indices. Panel B reports the variance inflation factor of each predictor in each Cox design matrix. Maximum absolute off-diagonal correlation is r=0.76; maximum VIF is 5.72 in the combined model and 5.20 in the primary Abdominal Fat Index model. Only L3 SATI and L3 VATI reach the moderate threshold (VIF>=5) and no predictor reaches the high threshold (VIF>=10). All three volumetric markers have VIF<5 in every model. This is the quantitative basis for the staged design with one volumetric marker per primary adjusted model.*

# Supplementary Figure S1. Diagnosis dates with the RAMIE cutpoint

*Supplementary Figure S1. Distribution of diagnosis dates across the 2011-2024 inclusion window. The dashed vertical line marks 1 July 2019, the institutional transition to robot-assisted minimally invasive esophagectomy (RAMIE), used as the era cutpoint in the sensitivity analyses (Supplementary Tables S2 to S5).*

# Supplementary Figure S2. Era-stratified Kaplan-Meier curves

*Supplementary Figure S2. Era-stratified Kaplan-Meier overall-survival curves comparing the Pre-RAMIE and RAMIE eras (cutpoint 1 July 2019). Panels show, in order, the overall cohort, the female and male strata, and the sex x M-status strata with sufficient sample size (female M0, male M0, male M1); each panel is titled with its stratum and reports n and events per era. The female metastatic stratum (n=4 in the RAMIE era) is omitted. Shaded bands are 95% confidence intervals and the log-rank p-values are those in Supplementary Table S3.*
